# Genomes of Two Flying Squid Species Provide Novel Insights into Adaptations of Cephalopods to Pelagic Life

**DOI:** 10.1016/j.gpb.2022.09.009

**Published:** 2022-10-07

**Authors:** Min Li, Baosheng Wu, Peng Zhang, Ye Li, Wenjie Xu, Kun Wang, Qiang Qiu, Jun Zhang, Jie Li, Chi Zhang, Jiangtao Fan, Chenguang Feng, Zuozhi Chen

**Affiliations:** 1 South China Sea Fisheries Research Institute, Chinese Academy of Fishery Sciences, Guangzhou 510300, China; 2 School of Ecology and Environment, Northwestern Polytechnical University, Xi'an 710072, China; 3 Southern Marine Science and Engineering Guangdong Laboratory (Guangzhou), Guangzhou 511458, China; 4 Key Laboratory for Sustainable Utilization of Open-Sea Fishery, Ministry of Agriculture and Rural Affairs, Guangdong Provincial Key Laboratory of Fishery Ecology and Environment, Guangzhou 510300, China; 5 Qinghai Provincial Key Laboratory of Crop Molecular Breeding, CAS Key Laboratory of Adaptation and Evolution of Plateau Biota, Northwest Institute of Plateau Biology, Chinese Academy of Sciences, Xining 810008, China; 6 CAS Key Laboratory of Aquatic Biodiversity and Conservation, Institute of Hydrobiology, Chinese Academy of Sciences, Wuhan 430072, China

**Keywords:** Cephalopoda, Evolution, Flying squid, Genome, Photophore

## Abstract

Pelagic cephalopods have evolved a series of fascinating traits, such as excellent visual acuity, high-speed agility, and **photophores** for adaptation to open pelagic oceans. However, the genetic mechanisms underpinning these traits are not well understood. Thus, in this study, we obtained high-quality **genomes** of two purpleback **flying squid** species (*Sthenoteuthis oualaniensis* and *Sthenoteuthis* sp.), with sizes of 5450 Mb and 5651 Mb, respectively. Comparative genomic analyses revealed that the *S*-crystallin subfamily *SL20-1* associated with visual acuity in the purpleback flying squid lineage was significantly expanded, and the **evolution** of high-speed agility for the species was accompanied by significant positive selection pressure on genes related to energy metabolism. These molecular signals might have contributed to the evolution of their adaptative predatory and anti-predatory traits. In addition, the transcriptomic analysis provided clear indications of the evolution of the photophores of purpleback flying squids, especially the recruitment of new genes and energy metabolism-related genes which may have played key functional roles in the process.

## Introduction

Cephalopods are a group of marine mollusk with remarkable morphology and behavior that play key ecological roles, are commercially important, and have been intensively studied [1]. Large populations of cephalopods inhabit depths ranging from shallow to abyssal [2], [3]. They are preyed upon by various apex predators (such as billfish, tuna, sharks, and cetaceans) that are generally the fastest and most efficient in the ocean [4], [5], [6]. In response to the predation, most cephalopods have developed excellent visual acuity [7], [8], [9], strong muscles, and morphological traits [10] that enable them to evade danger rapidly. They also have high metabolic levels and hence constantly high levels of energy supplies for their muscles [11]. The continuous adaptations and counter-adaptations induced by interactions between prey and predators — the ‘arms race’, is one of the most intense forms of co-evolution. This ‘arms race’ is particularly pronounced between the pelagic cephalopods (*e*.*g*., cuttlefishes and squids) and their predators [12]. These cephalopods have extremely strong muscles with obliquely-striated, quickly contractible, highly aerobic fibers, and morphological features, such as fins, which enable powerful swimming [10]. Some species of pelagic cephalopods have also repeatedly evolved photophores that assist in escape [13], predation [14], and mating [15]. However, although it has long been accepted that adaptative evolution has resulted in a series of fascinating traits in cephalopods [16], [17], [18], [7], the genetic mechanisms involved are much less clear.

Common pelagic cephalopods include members of the Ommastrephidae (Teuthida, Decapodiformes) called flying squids, which can jump out of the water and in some cases glide more than 30 m in the air [19]. They can achieve the fastest recorded speed (∼ 8 m/s) of any aquatic invertebrates [20], [21]. Important taxa with such capabilities include the purpleback flying squids (*Sthenoteuthis* spp.) [20], [22], [23]. These are the most abundant large squids in the tropical and subtropical Indo-Pacific ocean, found at depths from the surface to more than 600 m [2]. Moreover, the purpleback flying squids have a high degree of adaptation to their pelagic life and at least five morphological and ecological forms in terms of body size and possession or absence of photophores [2]. Thus, the purpleback flying squids are ideal models for studying the genetic mechanisms involved in the evolution of pelagic cephalopods’ adaptive traits.

**Supplementary Figure S1:**
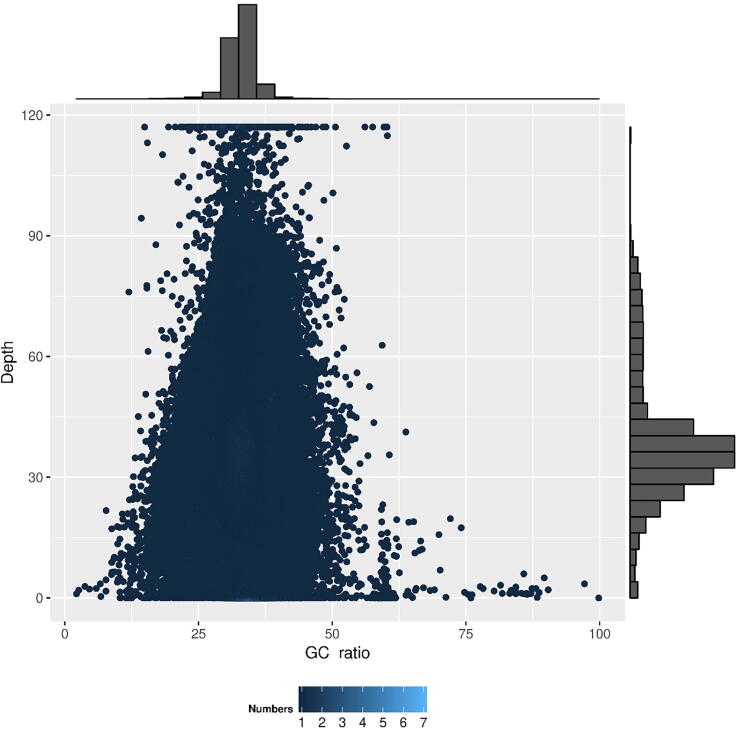
**Morphology and dissection of the two *Sthenoteuthis* species** “Typical form” and “dwarf form” of purpleback flying squids are referred to as *S. oualaniensis* and *Sthenoteuthis* sp., respectively. **A.** Morphology and the dorsal photophore patch of the “typical form”. **B.** Morphology and the corresponding position of the photophore (pseudo-photophore) of the “dwarf form”. **C.** Dissection of the female individual. **D.** Dissection of the male individual.

In this study, we constructed high-quality genomes for two ‘forms’ of purpleback flying squids. One (*S. oualaniensis*) is the ‘medium’ or ‘typical’ form, which has a dorsal mantel length (at maturity) of > 120 mm and spherical photophores forming an oval patch in the anterodorsal mantle musculature [2], [24] (Figure S1). The other (*Sthenoteuthis* sp.) is the ‘dwarf form’, which is smaller and lacks the dorsal photophore patch (Figure S1). The dwarf form was previously treated as *S. oualaniensis*, but is now considered an undescribed species [25], [26]. Through comparative genomic analyses, we deeply profiled genomic features of these two purpleback flying squids and investigated molecular signals associated with adaptations of pelagic life in cephalopods, such as their excellent visual system, high behavioral flexibility, and photophore. The results suggest that the genomes of these two purpleback flying squids are important resources that can facilitate research not only on cephalopods but also on adaptive evolution and molecular genetics more generally.

## Results and discussion

### Assemblies and genomic characteristics

**Figure 1:**
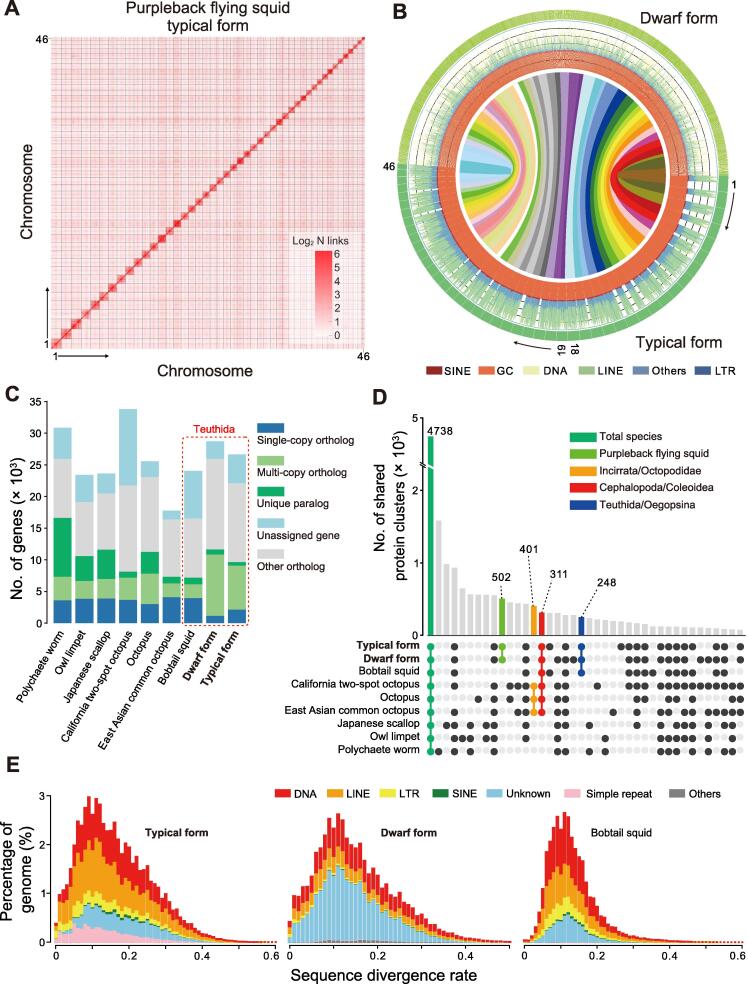
**Genome assemblies and annotations of two *Sthenoteuthis* species** *S. oualaniensis* and *Sthenoteuthis* sp. are referred to as the “typical form” and “dwarf form” of purpleback flying squids, respectively. **A.** Genome-wide Hi-C map of the 46 pseudo-chromosomes of *S. oualaniensis*. **B.** Syntenic comparison of *S. oualaniensis* and *Sthenoteuthis* sp. Numbers 1 to 46 refer to the chromosomes of *S. oualaniensis*. Each color block in the outermost layer represents a scaffold. Each line represents a syntenic block of five or more genes. Densities of specific kinds of TEs (ranging from 0 to 80%) were counted in 500-kb windows. **C.** Summary of gene clusters estimated from Orthofinder analysis based on sequences of eight Mollusca species and one annelid worm. **D.** UpSet plot of gene families. **E.** Divergence distribution of TEs of species in Teuthida. Latin binomials of the listed species are as follows: Polychaete worm, *Capitella teleta*; Owl limpet, *Lottia gigantea*; Japanese scallop, *Mizuhopecten yessoensis*; California two-spot octopus, *Octopus bimaculoides*; Octopus, *Octopus minor*; East Asian common octopus, *Octopus vulgaris*; Bobtail squid, *Euprymna scolopes*. TE, transposable element; LINE, long interspersed nuclear element; SINE, short interspersed nuclear element; LTR, long terminal repeat.

**Supplementary Figure S2:**
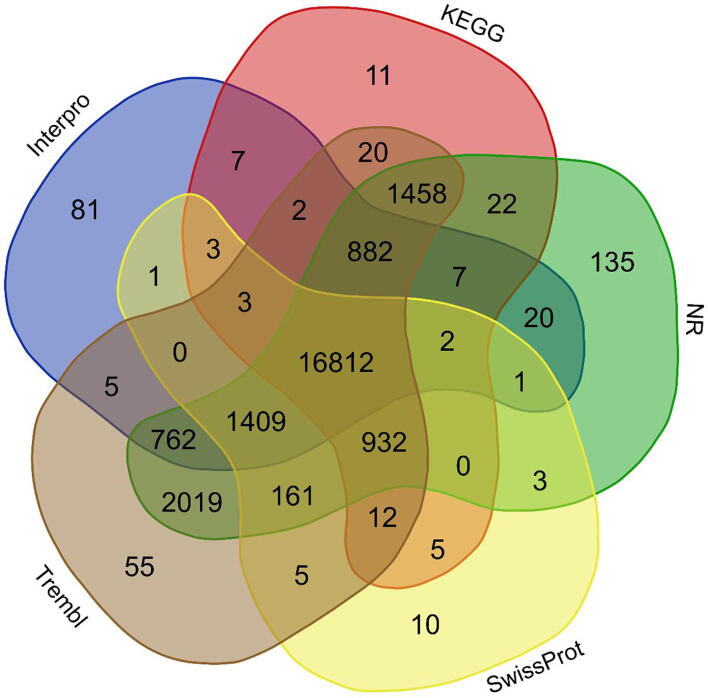
**Distribution of GC contents versus the sequencing depth for** A The horizontal and vertical coordinates indicate the GC contents and sequencing depth respectively with 10 kb as a window. The GC contents of most windows were around 20%–50%, indicating high-quality raw reads without significant contamination.

**Supplementary Figure S3:**
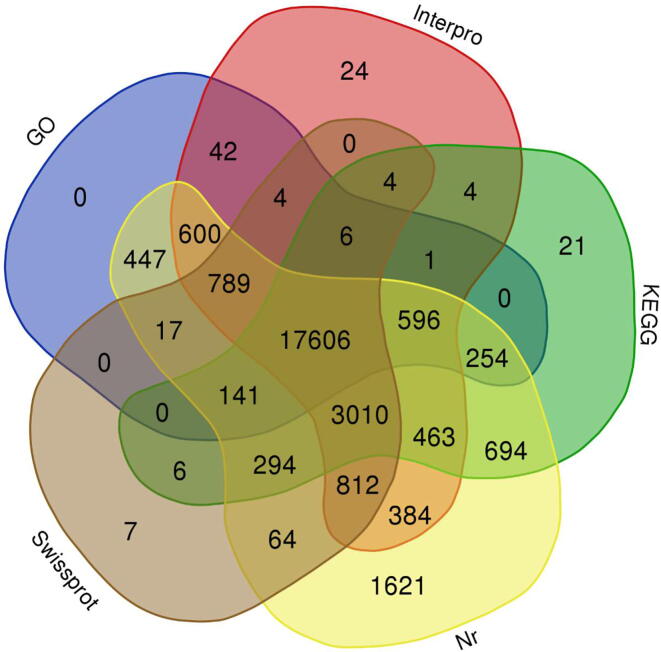
**Sequencing depth distribution for *S. oualaniensis*** The horizontal coordinate indicates the reads depth of PacBio reads. The vertical coordinate indicates the cumulative ratio of base depth. The blue line indicates the reads depth, the yellow line indicates the accumulation of all the reads depth.

**Supplementary Figure S4:**
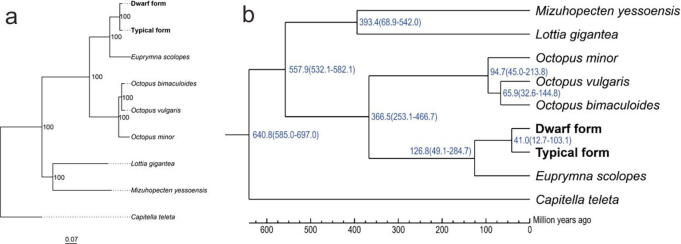
**GenomeScope profiles for *S. oualaniensis* were estimated** The result from jellyfish v2.2.6 with a *k*-mer size of 17 was used as the input file of GenomeScope v1.0. The second peak should be the major peak and has an estimated genome size of about 5.8 Gb. This is suggestive of the high heterozygosity of the species.

Using a combination of PacBio long reads, 10× Genomics short reads, RNA sequencing (RNA-seq), and Hi-C approaches, we obtained high-quality genomes of the two species of purpleback flying squids of the Ommastrephidae (*S. oualaniensis* and *Sthenoteuthis* sp.) (Tables S1–S4; Figures S2 and S3). The sizes of the genome assemblies were 5450 Mb and 5651 Mb for *S. oualaniensis* and *Sthenoteuthis* sp., respectively (Table 1), close to the genome sizes estimated by *k*-mer analysis (Figure S4). The *S. oualaniensis* genome was assembled to chromosome level, with 95.72% of contigs anchored to the 46 chromosomes (Figure 1A). The contig N50 and scaffold N50 values of the *S. oualaniensis* assembly were 1.52 Mb and 118.8 Mb, respectively, while the contig N50 value for the *Sthenoteuthis* sp. assembly was 1.3 Mb. The *S. oualaniensis* genome is the only squid genome that has been assembled to chromosome level (Figure 1A; Table 1). GC contents of the acquired *S. oualaniensis* and *Sthenoteuthis* sp. genomes were 33.70% and 33.00%, respectively, similar to those of other relatives (Table S5). Results of the Benchmarking Universal Single-Copy Orthologs (BUSCO) [27] assessment showed that the *S. oualaniensis* and *Sthenoteuthis* sp. assemblies had 89.8% and 93.6% genomic integrity, respectively (Table 1). RNA-seq analysis of *S. oualaniensis* and *Sthenoteuthis* sp. yielded 389,954 and 410,364 transcripts with total lengths of 200–25,632 bp and 200–31,105 bp, respectively (Table S6).

**Table 1:** **Summary of the assemblies and annotations of the two *Sthenoteuthis* species**

	***Sthenoteuthis oualaniensis***	***Sthenoteuthis* sp.**
Genome size (Mb)	5450	5651
Contig N50 (Mb)	1.52	1.3
Scaffold N50 (Mb)	118.8	-
GC content	33.07%	33.00%
Chromosome number	46	-
Gene number	26,646	28,715
BUSCO for genomes	89.4%	93.6%
BUSCO for genes	90.4%	94.3%

*Note*: BUSCO, Benchmarking Universal Single-Copy Orthologs.

**Supplementary Figure S5:**
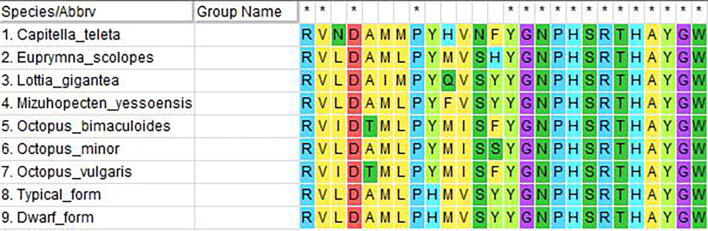
**Venn diagram of *S. oualaniensis* to show the well-aligned gene sets from a different database, including NR, InterPro, KEGG, SwissProt, and Trembl database**

**Supplementary Figure S6:**
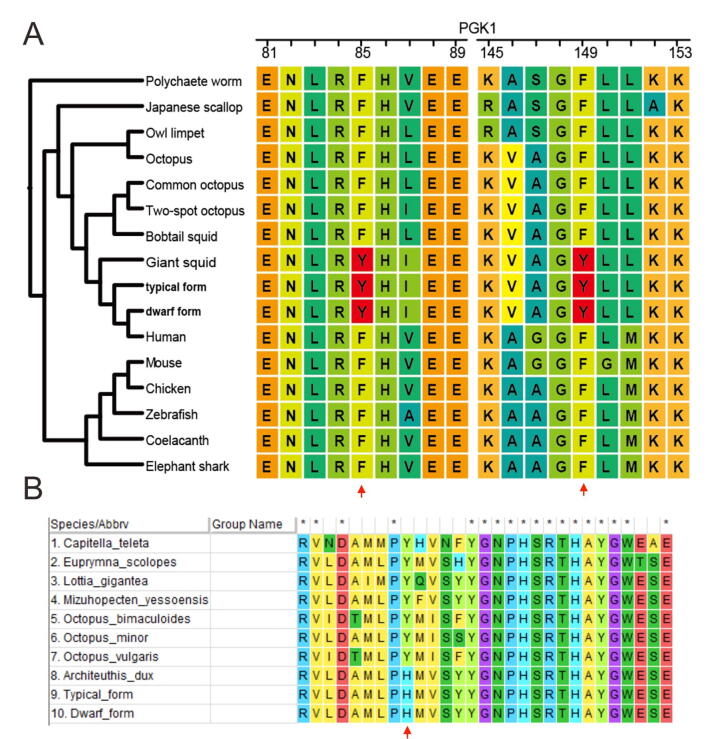
**Venn diagram of *Sthenoteuthis* sp. to show the well-aligned gene sets from a different database, including NR, InterPro, KEGG, SwissProt, and Trembl database**

Based on the high-quality assemblies, we predicted a total of 26,646 and 28,715 protein-coding genes in the genomes of *S. oualaniensis* and *Sthenoteuthis* sp., respectively (Table S5). Of these, 19,913 (74.73%) and 21,853 (76.10%) are supported by corresponding transcripts, and 24,845 (93.24%) and 27,897 (97.13%) predictions could be functionally annotated using entries in at least one database (Figures S5 and S6). Results of BUSCO analysis suggested that the assembly completeness of *S. oualaniensis* and *Sthenoteuthis* sp. were 89.4% and 93.6%, respectively, and the annotation completeness of them were 90.4 and 94.3%, respectively (Table 1, Table S7)*.* Basic metrics for the protein-coding genes of these two purpleback flying squids, including gene number/length, exon number/length, and codon usage, are close to those of other Mollusca (Table S5). Synteny analysis, based on the coding genes, identified 743 blocks with at least five syntenic genes between *S. oualaniensis* and *Sthenoteuthis* sp. and demonstrated the good colinearity between the two genomes, which also indicated that the two genomes were well assembled and annotated (Figure 1B). Finally, 22,162 (83%) *S. oualaniensis* genes and 25,991 (90%) *Sthenoteuthis* sp. genes were clustered into 16,235 gene clusters, with sizes close to those of other relatives (Figure 1C and D).

Repeats analysis indicated that close to half of the genomes of both *S. oualaniensis* and *Sthenoteuthis* sp. are composed of repetitive sequences: 53.90% and 42.37%, respectively (Tables S8 and S9), which is comparable to that of other cephalopods [28], [29]. Identification and classification of the transposable elements (TEs) of the two genomes (Figure 1E; Tables S8 and S9) showed that DNA and long interspersed nuclear element (LINE) were the two most abundant types of TEs in the *S. oualaniensis* genome (accounting for 25.47% and 22.05% of the total, respectively), but only accounted for 14.13% and 10.00% of the TEs, respectively, in the *Sthenoteuthis* sp. genome. There was a markedly smaller proportion of simple tandem repeats in the *S. oualaniensis* genome (6.33%) than in the *Sthenoteuthis* sp. genome (25.86%) (Tables S8 and S9). Thus, despite the very close relationship between the two purpleback flying squid species, they might have distinct patterns of TE activity.

### Phylogenetic status and species validity of two purpleback flying squids

**Figure 2:**
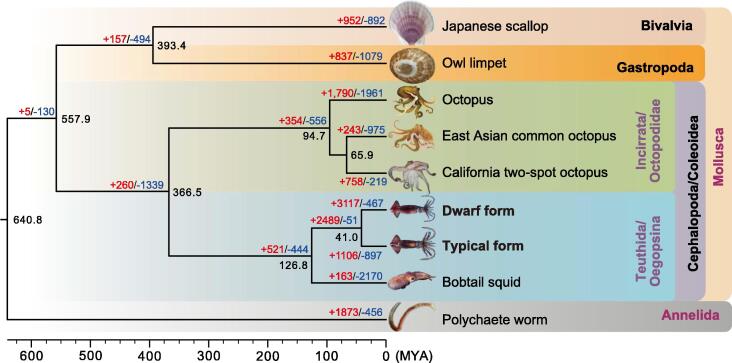
**Coalescence tree of eight Mollusca species and one annelid worm based on 334 single-copy orthologs** Estimated divergence time and expanded/contracted gene families are marked at the nodes. Red, blue, and black numbers indicate the number of expanded gene families, the number of contracted gene families, and the estimated divergence time, respectively. MYA, million years ago.

Maximum-likelihood (ML) gene tree and species tree analyses based on 334 one-to-one genes yielded consistent topologies (Figure S7). The Octopodiformes and Decapodiformes species each clustered into monophyletic lineages, which apparently diverged around 366.5 million years ago (MYA) (Figure 2; Figure S7). The two purpleback flying squid species are the most closely related of the included taxa, having diverged at approximately 41.0 MYA. These two forms of purpleback flying squids, *S. oualaniensis* and *Sthenoteuthis* sp., were considered to be members of the same species for a long time, but this was recently questioned [25], [26]. Morphological studies provided evidence that *Sthenoteuthis* sp. was distinguishable from *S. oualaniensis* concerning external features, including variables of the head, carcass, and arms, as well as the shape and size of fins [30], [31]. Our results corroborate the conclusion that *Sthenoteuthis* sp. should be regarded as a distinct species, as we detected clear differences in their genomes and derived a substantial divergence time.

### Excellent vision of purpleback flying squids

Keen vision is regarded as a major product of evolutionary arms races in squids [7], which strongly contributes to the ability of purpleback flying squids (and others) to avoid predation and catch prey even in dim conditions [1], [7], [9]. Important components of their eyes include several soluble crystallins that play key roles in maintaining the transparency and optical clarity of the lens [32]. In particular, *S*-crystallins are present in the lenses of many cephalopods, and have refractive properties that strongly contribute to good vision (and hence cephalopod survival) in poor light [33], [34]. *S*-crystallins are even claimed to provide a “perfect medium”, forming gels of varying density, in the spherical lenses of cephalopods [34], [35], [36].

**Figure 3:**
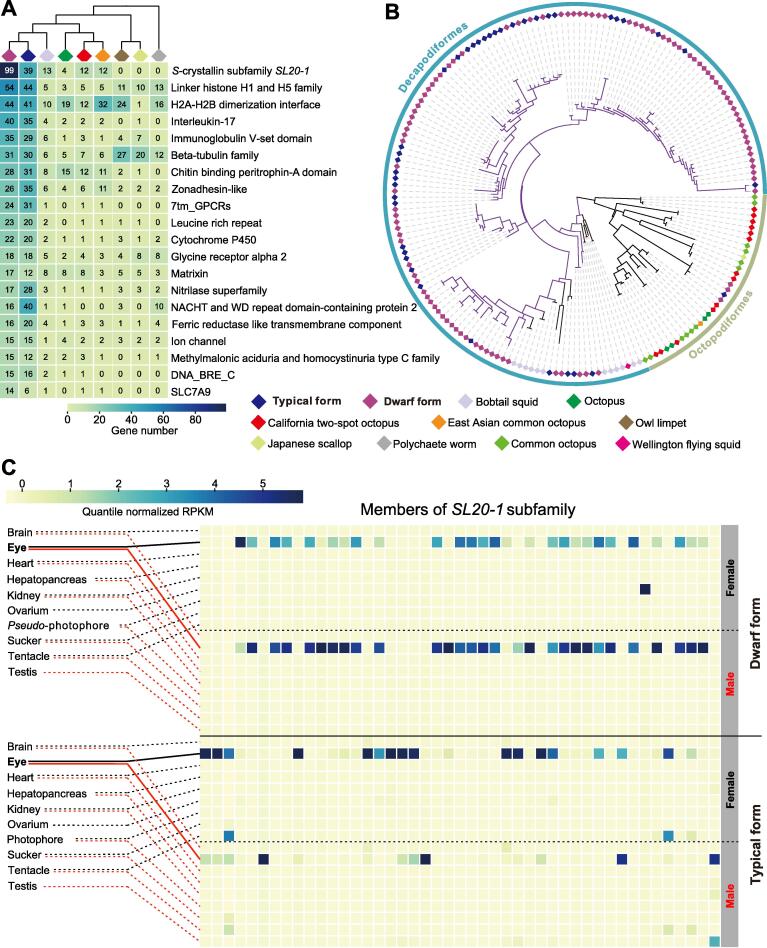
**Expanded gene families in the *Sthenoteuthis* lineage** **A.** The top 20 expanded gene families. **B.** Unrooted maximum-likelihood tree of the massive expansion of the *S*-crystallin subfamily *SL20-1*, with 39 and 99 gene members in *S. oualaniensis* and *Sthenoteuthis* sp., respectively. **C.** Expression patterns of coexisting gene members of the *SL20-1* subfamily in *S. oualaniensis* and *Sthenoteuthis* sp. Only those expressed gene members were shown. These gene IDs were detailed in Figure S10. Most of the significantly expanded genes were highly expressed in the eyes. Commen octopus, *Octopus sinensis*; Wellington flying squid, *Sepia pharaonis*.

Cluster analysis detected 2489 gene families that were expanded in both purpleback flying squid species (under the criterion of both family-wide and viterbi *P* values < 0.01; Figure 2, Figures S8 and S9). The *SL20-1* gene subfamily which encodes *S*-crystallins was most significantly expanded, with 39 and 99 gene members in *S. oualaniensis* and *Sthenoteuthis* sp., respectively (Figure 3A and B). Transcriptomic analysis revealed that most of these expanded *SL20-1* genes were only highly expressed in eyes (Figure 3C, Figure S10), clearly indicating that expansion of this subfamily plays an important role in the emergence of purpleback flying squids’ excellent vision.

### High behavioral flexibility

The mobility of organisms strongly affects their predatory and anti-predatory abilities, and thus their evolutionary fitness [37]. Purpleback flying squids are highly successful in these terms, as they are the fastest and most mobile aquatic invertebrates, with very high metabolic levels that enable powerful output at all times [11], [23].

**Figure 4:**
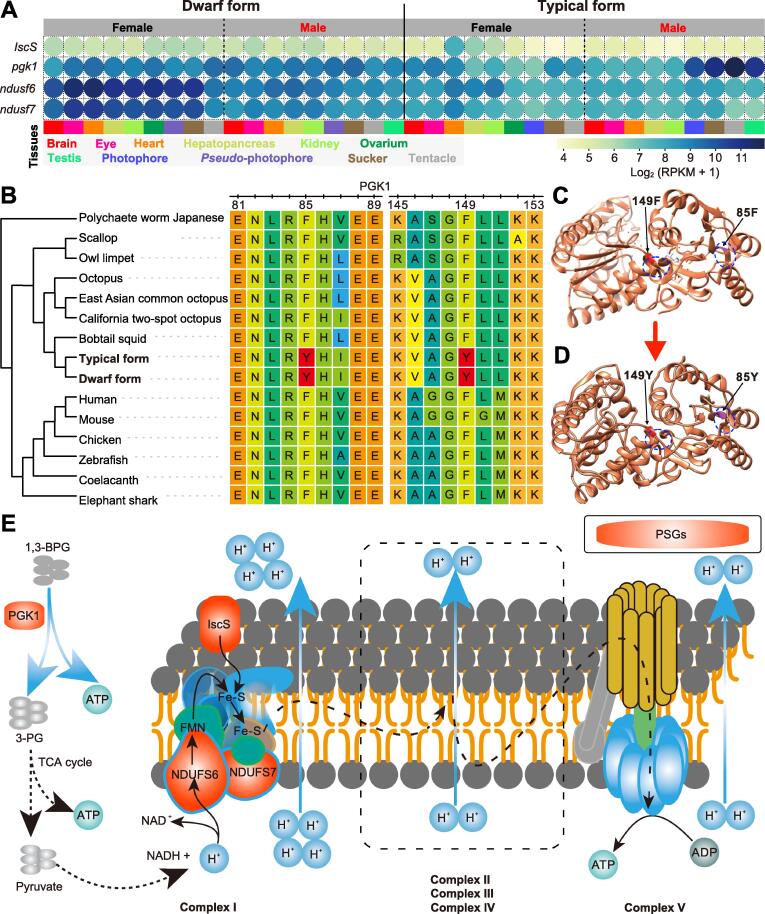
**Diagram ofPSGsassociated with energy metabolism** **A.** Expression patterns of four PSGs involved in energy metabolism that are highly expressed in all investigated tissues. **B.** Positively selected signals in two extremely conserved regions of PGK1. **C.** Three-dimensional structure of mouse PGK1 protein downloaded from the PDB database. Substructures of 85F and 149F are highlighted. **D.** Three-dimensional structure simulated by a homologous approach of mouse PGK1 with F85Y and F149Y substitutions. Structures adjacent to the substitute sites in (C) and (D) are signaled by the blue dashed circles. **E.** Schematic diagram of the glycolysis pathway and respiratory electron chain. PSG, positively selected gene; PGK1, phosphoglycerate kinase 1; PDB, Protein Data Bank; RPKM, reads per kilobase million.

Our analysis identified 66 positively selected genes (PSGs) in the *Sthenoteuthis* lineage [false discovery rate (FDR)-adjusted *P* < 0.01; Table S10]. Gene Ontology (GO) enrichment analyses (FDR < 0.01) indicated that 23, 42, and 30 of these PSGs were associated with cellular components, molecular functions, and biological processes, respectively (Figures S11 and S12). The PSGs involved in energy metabolism were apparently under significant evolutionary selection pressure (FDR-adjusted *P* < 0.01). “Phosphoglycerate kinase activity” (GO:0004618, *e.g.*, *pgk1*) and “[2Fe-2S] cluster assembly” (GO:0044571, *e.g.*, *IscS*) were the two most significant GO terms (FDR < 0.05; Tables S11 and S12). The “quinone binding” term (GO:0048038, *e.g.*, *ndufa6* and *ndufa7*) was also significantly enriched (FDR < 0.05; Table S11). Transcriptomic analysis demonstrated that these genes were highly expressed in all the investigated tissues, implying that they play important roles (Figure 4A).

Phosphoglycerate kinase 1 (PGK1), the first ATP-generating enzyme in the glycolytic pathway, both directly generates ATP and indirectly supplies fuel for the mitochondrial electron respiratory chain [38], [39]. Phylogenetic analysis by maximum likelihood (PAML) detected positive selection signals at two extremely conserved regions of the *pgk1* gene (F85Y and F149Y; Figure 4B). Simulations showed that these two F-to-Y substitutions affect the structure of PGK1 protein resulting in the conversion of the original helix to a loop (Figure 4C and D). Thus, these substitutions were presumably highly important for purpleback flying squids. Similarly, a positive selection signal was detected at a conserved region of the *IscS* gene (Y67H; Figure S13). *IscS* participates in the synthesis of multiple iron-sulfur (Fe-S) proteins and the formation of Fe-S clusters in complex I (NADH: ubiquinone oxidoreductase; Figure 4E) [40]. Knockdown of the *IscS* gene leads to a decrease in mitochondrial activity [40], [41]. We also found positive selection signals in *ndufa6* and *ndufa7*, which encode two important subunits of complex I that are essential for the catalytic activity of this complex (Table S10) [42], [43]. Complex I plays a key role in ATP synthesis driven by the mitochondrial electron respiratory chain [44] (Figure 4E). Therefore, the PSGs mentioned above are likely to promote ATP synthesis, which is important for the maintenance of high metabolic levels and the high behavioral flexibility of purpleback flying squids. Furthermore, the presence of these positively selected sites in the pelagic *Architeuthis dux* (Figure S14), which also possess high metabolic levels [2], [29], implies the importance of these selection signals for pelagic cephalopods. However, we should note that these results come from a small gene pool and therefore have some limitations.

### Photophore transcriptome

**Figure 5:**
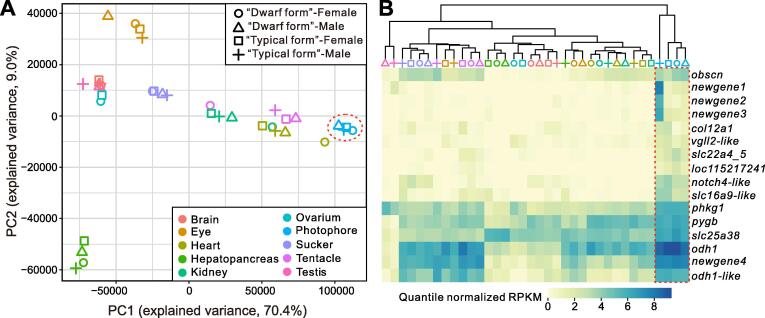
**Results of photophore transcriptome analysis** **A.** PCA plot of 36 RNA-seq samples based on expression profiling of 23,082 orthologs. *Pseudo*-photophores of *Sthenoteuthis* sp. and photophores of *S. oualaniensis* clustered together (red dotted circle). **B.** Whole-tissue expression patterns of 16 highly expressed genes in the four “photophore” tissues. PCA, principal component analysis; PC, principal component.

Bioluminescence is a common feature of cephalopods, especially pelagic species [14], [45]. At least 63 genera of squid and cuttlefish have repeatedly evolved photophores that play important roles in defense, predation, and communication [13], [14], [15]. One of the species included in this study, *S. oualaniensis*, has a dorsal photophore patch, but not *Sthenoteuthis* sp. (Figure S1). Therefore, they are ideal models for studying the evolution of photophores. Principal component analysis (PCA) showed that *S. oualaniensis* photophores clustered with the *pseudo*-photophores (‘muscle tissue’ corresponding to the position of the photophore of *S. oualaniensis*) of *Sthenoteuthis* sp. (Figure 5A), and highly expressed genes of photophores had similar expression patterns (Figure 5B). Therefore, the *pseudo*-photophores of *Sthenoteuthis* sp. and photophores of *S. oualaniensis* seem to be homologous organs, and the *pseudo*-photophores may have some essential functions similar to those of the photophores.

Previous studies based on gene expression profiles have suggested that the massive recruitment of pre-existing gene modules plays an important role in the formation of cephalopod photophores [46], [47]. Here we specifically investigated the highly expressed genes in the “photophores” of these two squids, which represent genetic factors associated with the essential function of this organ. Among the 16 highly expressed genes, four were new genes (*newgene1*–*4*; Figure 5B). Although functional assignations of these genes were not supported by homologs in public databases, their specific expression patterns suggest that they might participate in the formation and some of the basic functions of luminophores. In addition, four of the highly expressed genes (*phkg1*, *pygb*, *odh1*, and *obh1*-like) are energy metabolism-related genes. All of these genes are involved in glucose metabolism. Both *phkg1* and *pygb* are glycogen phosphorylases that regulate the catabolism of glycogen and provide glucose used in glycolysis [48], [49]. Octopine dehydrogenase (encoded by *odh1*), mainly found in mollusks [50], has a major function similar to that of lactate dehydrogenase, providing an important reducing agent for the glycolytic process [51]. These observations imply that these new genes and recruited energy metabolism-related genes might provide important support for the photophores of purpleback flying squids.

## Conclusion

In this study, we generated high-quality genomes of the two purpleback flying squid species. Comparative genomic analyses indicated that expansion of the *S*-crystallin subfamily *SL20-1* and locus variation and expression patterns of genes related to energy metabolism are associated with adaptations of purpleback flying squids (such as excellent vision, high behavioral flexibility, and photophore) that have played important roles in the ‘arms race’ and other pelagic adaptations among marine organisms. Moreover, the study supports the validity of treating *Sthenoteuthis* sp. as a separate species at the genomic level. These findings advance our understanding of the genetic basis of pelagic cephalopods associated with predatory and anti-predatory traits and suggest that the two genomes could be important resources for studying not only cephalopods, but also co-evolution, bioluminescence, and other broader aspects of molecular genetics.

## Materials and methods

### Sampling and sequencing

Squid samples were caught by a commercial fishing vessel using a lit falling net at night in the South China Sea. One male individual and one female individual of both medium (*S. oualaniensis*) and dwarf (*Sthenoteuthis* sp.) purpleback flying squids were collected for sequencing. Muscles from the males were used for DNA extraction and genomic library preparation. A PacBio Sequel device was used for sequencing the long reads. Short-insert paired-ends libraries were prepared and sequenced according to the Illumina sequencing protocol. Sample indexing and partition barcoded libraries were prepared using a chromium genome reagent kit (Catalog No. PN-120229, 10× Genomics, CA) and sequenced by an Illumina HiSeq X-Ten system for Hi-C analysis of *S. oualaniensis*. To explore gene expression patterns of the species and aid gene annotation, RNA was extracted from nine tissues, including tentacle, brain, eye, heart, kidney, sucker, hepatopancreas, ovarium/testis, and photophore (for *Sthenoteuthis* sp., muscle tissue corresponding to the position of the photophore of *S. oualaniensis* was obtained), from each of the four *S. oualaniensis* and *Sthenoteuthis* sp. individuals for library preparation and sequenced using the Illumina HiSeq 2000 platform.

### Estimation of genome sizes of the two ***Sthenoteuthis*** species

The genome sizes of the two *Sthenoteuthis* species were estimated by *k*-mer analysis using filtered Illumina reads. We used SOAPec v2 [52] to estimate the distribution of 17-mer depth, and then estimated the genome size from the total base and peak values of 17-mer depth.

### Genome assembly

Based on the estimated genome sizes, we first assembled genomes of the two purpleback flying squid species to contig level with wtdbg2 v2.4.1 [53] and standard parameters. The arrow algorithm was used to polish the two draft genomes with the filtered PacBio reads. Then the filtered short paired-end reads were aligned to the draft genome by BWA-MEM v.0.7.12-r1039 [54] with standard parameters, and Pilon [55] was used to further polish the genomes in two rounds using the sorted bam files. Finally, the genome assembly of *S. oualaniensis* was anchored with the Hi-C reads by 3D-DNA [56] and Juicer v1.5 [57]. To improve the quality of the chromosome assembly, we used Juicebox assembly tools [58] to remove potential assembly errors. BUSCO v3.02 [27] with the “metazoa_odb9” library was used to evaluate the completeness of the two assemblies.

### Repetitive sequence annotation

After obtaining a high-quality genome assembly, we used a combination of *de novo* and homologous predictions to annotate repetitive sequences, including tandem repeats and TEs. Firstly, tandem repeat finder v4.07 [59] was used to scan the tandem repeat elements with the parameter settings “2 7 7 80 10 50 500 -d -h -ngs”. Then we used RepeatModeler v1.0.8 [60] to build a *de novo* repeat library, and RepeatMasker v3.3.0 [61] to detect homologous repeat elements. After integrating the results of *de novo* and homologous predictions, Jukes-Cantor distances were calculated, and the R8s algorithm [62] was used to calculate rates of evolution from them.

### Protein-coding gene prediction

A combination of *ab initio*, homologous, and transcript-based gene predictions was used to integrate the two genomes. The gene prediction pipeline was as follows. First, AUGUSTES v3.2.1 [63], GlimmerHMM v3.02 [64], and GeneID v1.4 [65] were used for *de novo* gene prediction. Second, we downloaded the non-redundant proteomes of *Lottia gigantea* (GCF_000327385.1), *Mizuhopecten yessoensis* (GCF_002113885.1), *Octopus bimaculoides* (GCF_001194135.1), *Euprymna scolopes* (GCA_004765925.1), *O. minor* (http://dx.doi.org/10.5524/100503), and *O. vulgaris* (GCA_003957725.1) for homologous gene prediction. We used TBLASTN v2.9 [66] to align the proteomes of the six relatives to the two purpleback flying squid genomes and extended 10,000 bp in both directions from the start and end of every TBLASTN hit. Then, all non-redundant transcripts of all tissues were aligned to the genome with TBLASTN and a 1000-bp extension was applied. Genewise v2.4 [67] was then used to resolve the gene structure according to the aforementioned hits. Next, we integrated results of the *ab initio*, homologous, and transcript-based predictions with 1:4:5 weights using EvidenceModeler [68]. Finally, for further functional annotation of these two gene sets, we scanned public databases, including Swiss-Prot, KOG, Nr, KEGG, GO, and Pfam to detect the best matches using Interproscan v5 [69].

### Gene family clustering analysis

In addition to the two predicted gene sets and six Mollusca species mentioned above, we downloaded the genome of *Capitella teleta* (GCA_000328365.1) as an outgroup. Proteomes of these nine species were subjected to an all-vs-all BLAST search (E-value ≤ 1E−6) and then clustered by OrthoFinder [70] with default parameters. The shared gene clusters were visualized by the R package UpSetR [71]. The expanded and contracted gene families were investigated by CAFÉ v4.0.1 [72] using the result of the clustering analysis under the criterion of both family-wide and viterbi *P* values < 0.01.

### Phylogeny and divergence time estimation

Based on the cluster analysis of the aforementioned nine species, the protein-coding sequences and corresponding codon sequences of 334 one-to-one homologous genes were picked out and aligned using MAFFT v7 [73]. The bad alignments were removed by trimAl [74]. Finally, we used RAxML v8.2.4 [75] with “-m GTRGAMMA -f a -x 271828 -N 100 -p 54321” parameter settings to construct phylogeny trees and ASTRAL [76] to infer a species tree. MCMCtree in the PAML package [77] was used to estimate divergence times in conjunction with two softbound calibration points from https://www.timetree.org, *O. bimaculoides*–*C. teleta* (585–679 MYA) and *O. bimaculoides*–*L. gigantea* (531–582 MYA).

### Synteny between the genomes of two *Sthenoteuthis* species

To evaluate the conservation and quality of the two assemblies of *Sthenoteuthis* species, we used the *S. oualaniensis* assembly as a reference and *Sthenoteuthis* sp. assembly as the query in alignment analysis by LAST v942 [78] with the “-E 0.05” parameter setting. We also calculated the densities of repetitive elements and GC contents in 500-kb windows of the genomes. Finally, the results of these analyses were integrated into a circular layout by CIRCOS v0.69 [79].

### Positive selection analysis

To evaluate the evolutionary pressure on *Sthenoteuthis*, we used the one-to-one homologous genes of the nine species listed above (see the “Gene family clustering analysis” section for details) to identify PSGs. We aligned codons of all the one-to-one homologous genes by PRANK v140603 [80] with “-codon -f = fasta”. All the gaps generated by alignments were removed by Gblocks v0.91b [81] with “-t = c”. Then we used an in-house Perl script to convert the aligned sequence to PAML format for use in PAML analysis. Finally, PAML 4.9i [77] was used to analyze the selection pressure on each gene with the ML method under the branch-site model. A species tree constructed from ASTRAL analysis was used as the input tree. The two *Sthenoteuthis* species were selected as foreground and the other seven species as background. The significance of the alternative model (estimated omega) against the null model (fixed omega) was assessed by likelihood ratio tests (LRTs), in which twice the log-likelihood difference (2DL) values were calculated and compared to a chi-squared distribution. Genes with *P* < 0.01 (with FDR correction) and carrying at least one site under positive selection with a Bayes empirical Bayes (BEB) posterior probability > 0.8, were identified as candidate PSGs.

### Protein structure simulation

A homology-based approach was used to simulate structures of proteins encoded by PSGs. We first sought matches to the *S. oualaniensis* PGK1 protein sequence in the Protein Data Bank (PDB) database (https://www.rcsb.org/) and selected the hit with the highest score as a potential template for the simulation of the PGK1 protein structure. The corresponding positively selected sites (F85 and F149) of mouse PGK1 protein were replaced by those of *Sthenoteuthis* (85Y and 149Y). Then, the modified sequence was submitted to Phyre2 [82] for structure simulation. Finally, the Phyre2 result with the highest score was selected as the final structure and visualized by UCSF Chimera [83].

### Transcriptomic analysis

Raw reads obtained from RNA-seq of the nine mentioned tissues of the four sampled individuals were filtered using fastp [84] with default parameters. The low-quality reads were removed by Sickle v1.33 (https://github.com/najoshi/sickle) with default parameters except for the “pe” setting. The cleaned reads were mapped to the reference genome with HISAT2 [85]. Then Trinity [86] was used to assemble these reads into transcripts. Next, we used TransDecoder [87] with default parameters to predict gene structures of the transcripts and CD-HIT [88] to remove redundant predictions. The numbers of reads and reads per kilobase of transcript per million mapped reads (RPKM) values for all genes in the 36 tissues were calculated by StringTie v2.1.4 [89] using the output of HISAT2 [85] analysis. Each gene with an RPKM value greater than 1 was considered a validly expressed gene. The *Tau* value of genes in all tissues was calculated using an in-house Perl script. Genes expressed in the photophore with *Tau* values ≥ 0.8 and higher RPKM values than in other tissues were regarded as being highly specifically expressed in the photophore. All the RNA-seq data for the 36 tissues were subjected to PCA, using normalized and logarithmically transformed RPKM values. Seaborn [90] was used to cluster and visualize clusters of the highly expressed genes in the photophore. Those genes shared only by the two *Sthenoteuthis* species and supported by transcripts were identified as new genes. To investigate the expression pattern of expanded genes of the *SL20-1* subfamily across organs, RNA-seq reads of both species were mapped to the genomes of *Sthenoteuthis* sp. and subsequently assessed by RPKM values.

## Code availability

The custom scripts related to this work have been deposited in BioCode at the National Genomics Data Center (NGDC), Beijing Institute of Genomics (BIG), Chinese Academy of Sciences (CAS) / China National Center for Bioinformation (CNCB), and are publicly accessible at https://ngdc.cncb.ac.cn/biocode/tools/BT007297/releases/1.0.

## Data availability

The genomes and annotations of these two purpleback flying squid species, raw sequencing data, and acquired RNA-seq data have been deposited in the China National GeneBank (CNGB: CNP0001958), and are publicly accessible at https://db.cngb.org/cnsa. The raw sequencing data have also been deposited in the Genome Sequence Archive [91] at the NGDC, BIG, CAS / CNCB (GSA: CRA004867), and are publicly accessible at https://ngdc.cncb.ac.cn/gsa. The genomes and annotations of these two purpleback flying squid species have also been deposited in the Genome Warehouse [92] at the NGDC, BIG, CAS / CNCB (GWH: GWHBECU00000000 for *S. oualaniensis*; GWHBFHL00000000 for *Sthenoteuthis* sp.), and are publicly accessible at https://ngdc.cncb.ac.cn/gwh.

## CRediT author statement

**Min Li:** Conceptualization, Validation, Formal analysis, Investigation, Project administration, Writing - original draft. **Baosheng Wu:** Formal analysis, Data curation, Visualization, Writing - original draft. **Peng Zhang:** Validation, Resources, Investigation, Writing - review & editing. **Ye Li:** Formal analysis, Data curation, Visualization. **Wenjie Xu:** Formal analysis, Data curation, Visualization. **Kun Wang:** Writing - review & editing. **Qiang Qiu:** Writing - review & editing. **Jun Zhang:** Resources, Writing - review & editing. **Jie Li:** Resources, Investigation. **Chi Zhang:** Methodology, Software, Data curation. **Jiangtao Fan:** Resources, Writing - review & editing. **Chenguang Feng:** Conceptualization, Methodology, Validation, Project administration, Writing - original draft. **Zuozhi Chen:** Supervision, Project administration, Funding acquisition, Writing - review & editing. All authors have read and approved the final manuscript.

## Competing interests

The authors have declared no competing interests.

## Supplementary material

Supplementary Figure S7 **Estimation of phylogeny and divergence times among purpleback flying squid species and their relatives** The divergence time was indicated by the blue number near each node, while the 95% CI was indicated by the blue brackets and its specific value was colored in blue under the corresponding divergence time. Two softbound calibration time points had been applied: Octopus bimaculoides – *Capitella teleta* (585–679 MYA) and *Octopus bimaculoides – Lottia gigantea* (531–582 MYA).

Supplementary Figure S8 **Top 20 KEGG enriched pathway of the significantly expanded gene families for both *Sthenoteuthis* species**

Supplementary Figure S9 **The significantly enriched GO terms of the expanded gene families for both *Sthenoteuthis* species**

Supplementary Figure S10 **Expression patterns of coexisting gene members of the *SL20-1* subfamily in *S. oualaniensis* and *Sthenoteuthis sp.*** Only those expressed gene copies were shown. Gene IDs and tissue information correspond to Figure 3C. Most of the significantly expanded genes were highly expressed in the eyes.

Supplementary Figure S11 **Classification of the three major GO function annotations for the 66 PSGs of the two *Sthenoteuthis* lineage**

Supplementary Figure S12 **The significantly enriched KEGG pathway of the positively selected genes for both *Sthenoteuthis* species**

Supplementary Figure S13 **Sequence alignments of *IscS* for the two *Sthenoteuthis* and other seven species from Mollusca and Annelida**

Supplementary Figure S14 **Positive selection sites for genes PGK1 (Figure 4B) and *IscS* (Figure S13) shared by the two *Sthenoteuthis* were also detected from the giant squid (*Architeuthis dux*)**
