# Supplementary figures and images for: Genomes of Two Flying Squid Species Provide Novel Insights into Adaptations of Cephalopods to Pelagic Life
Source: Genomics Proteomics Bioinformatics. 2022 Oct 7;20(6):1053–65. doi: 10.1016/j.gpb.2022.09.009 (PMC10225486; doi:10.1016/j.gpb.2022.09.009)

**A**

Photophore

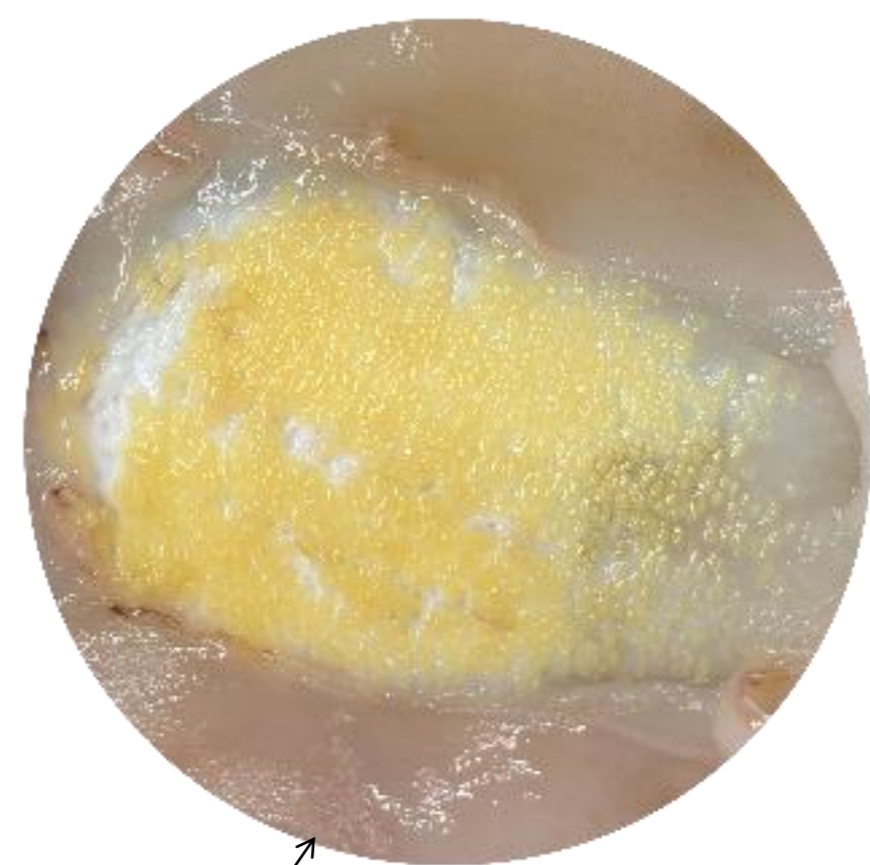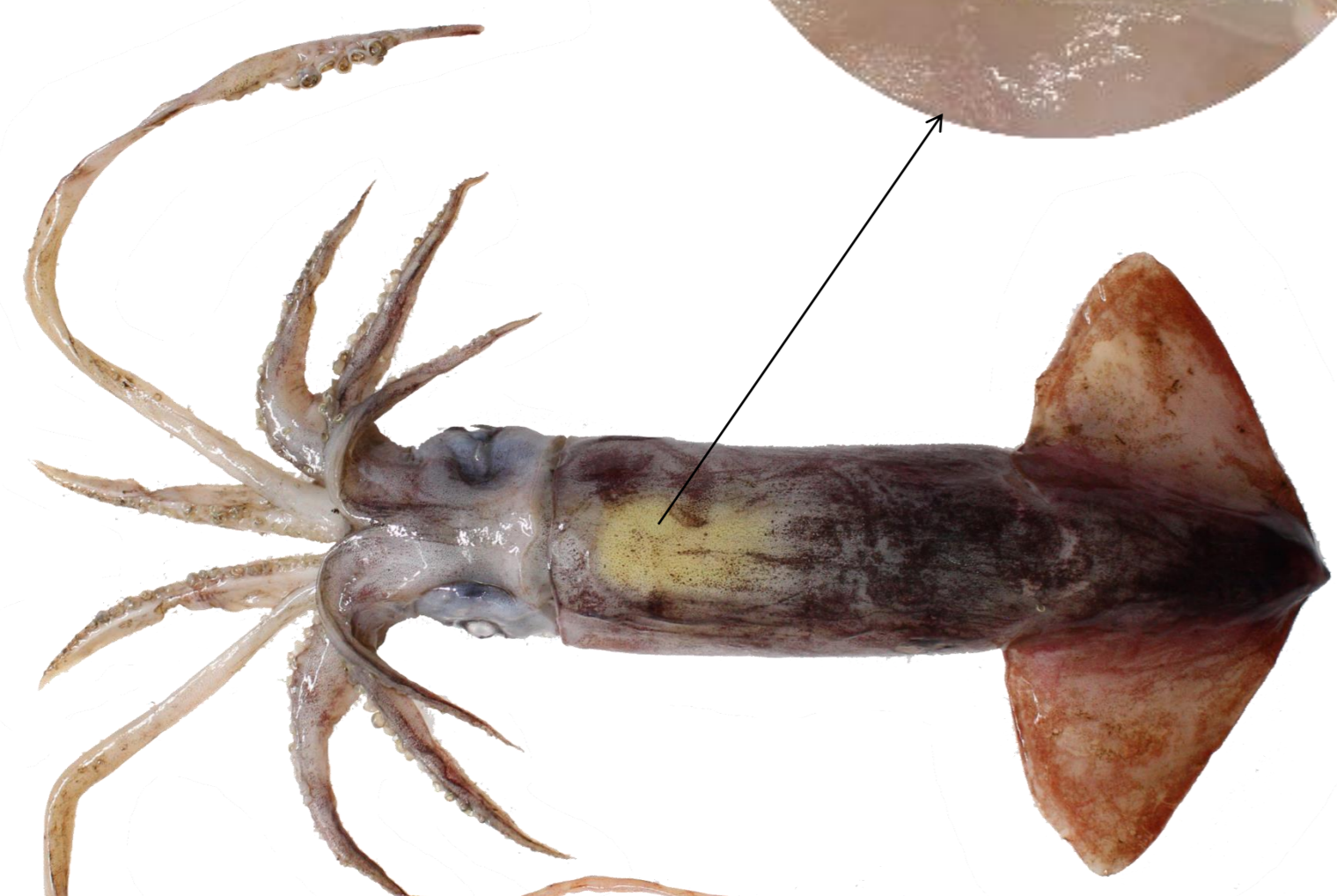

**B**

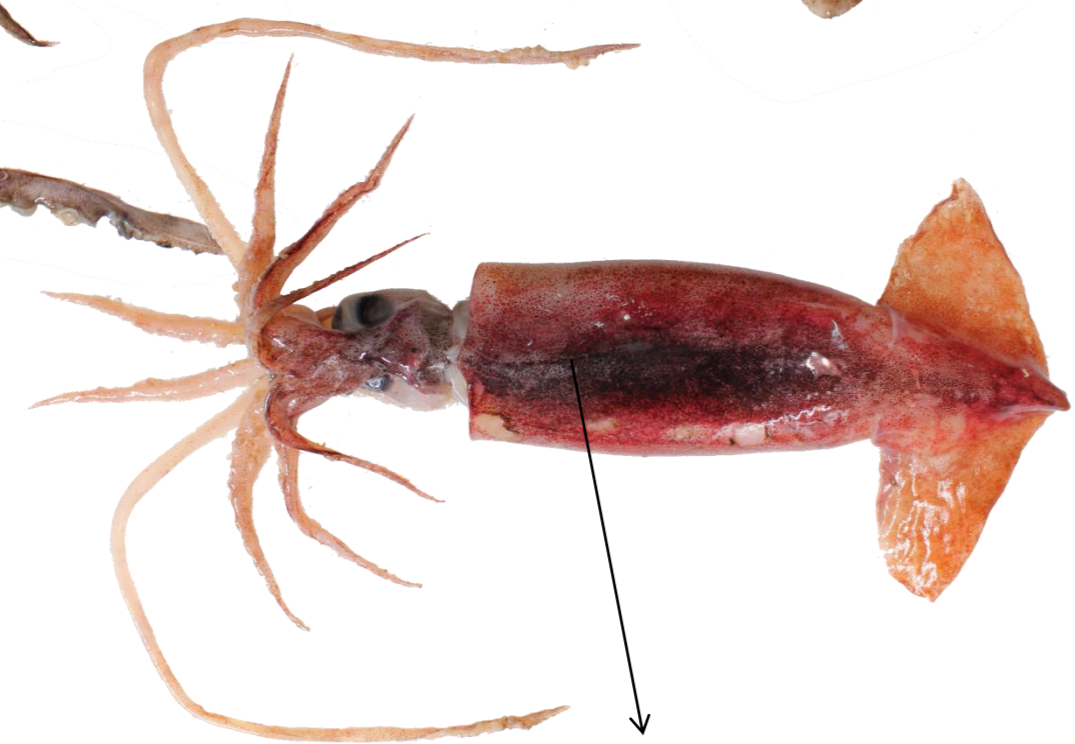

*pseudo*-Photophore

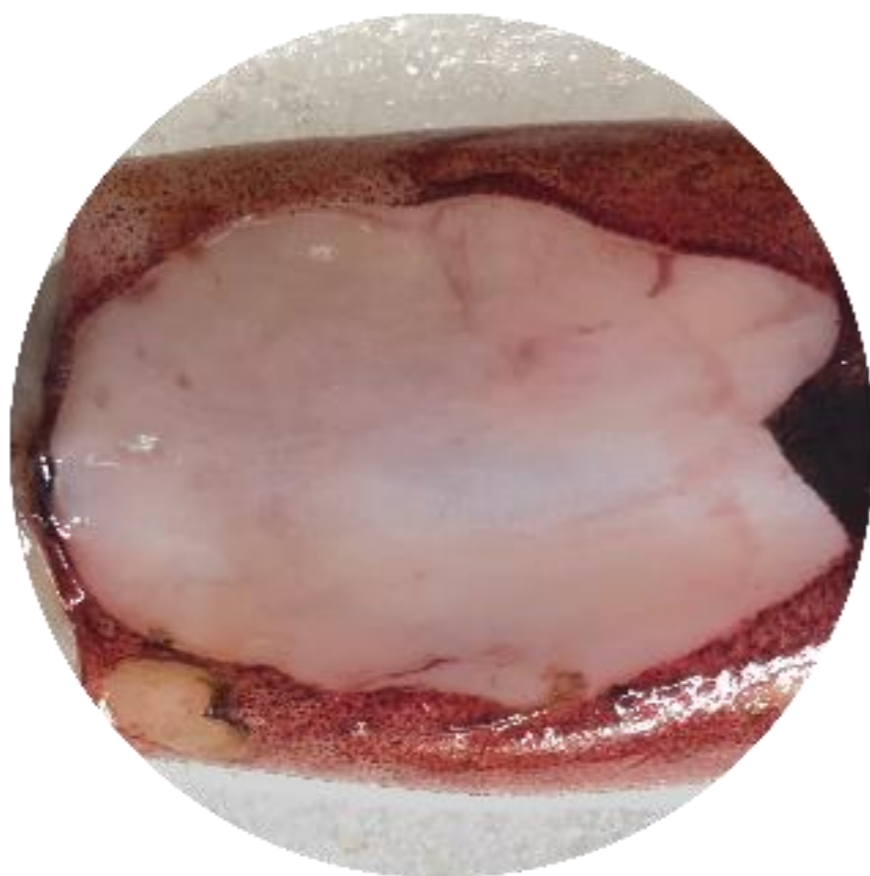

**C**

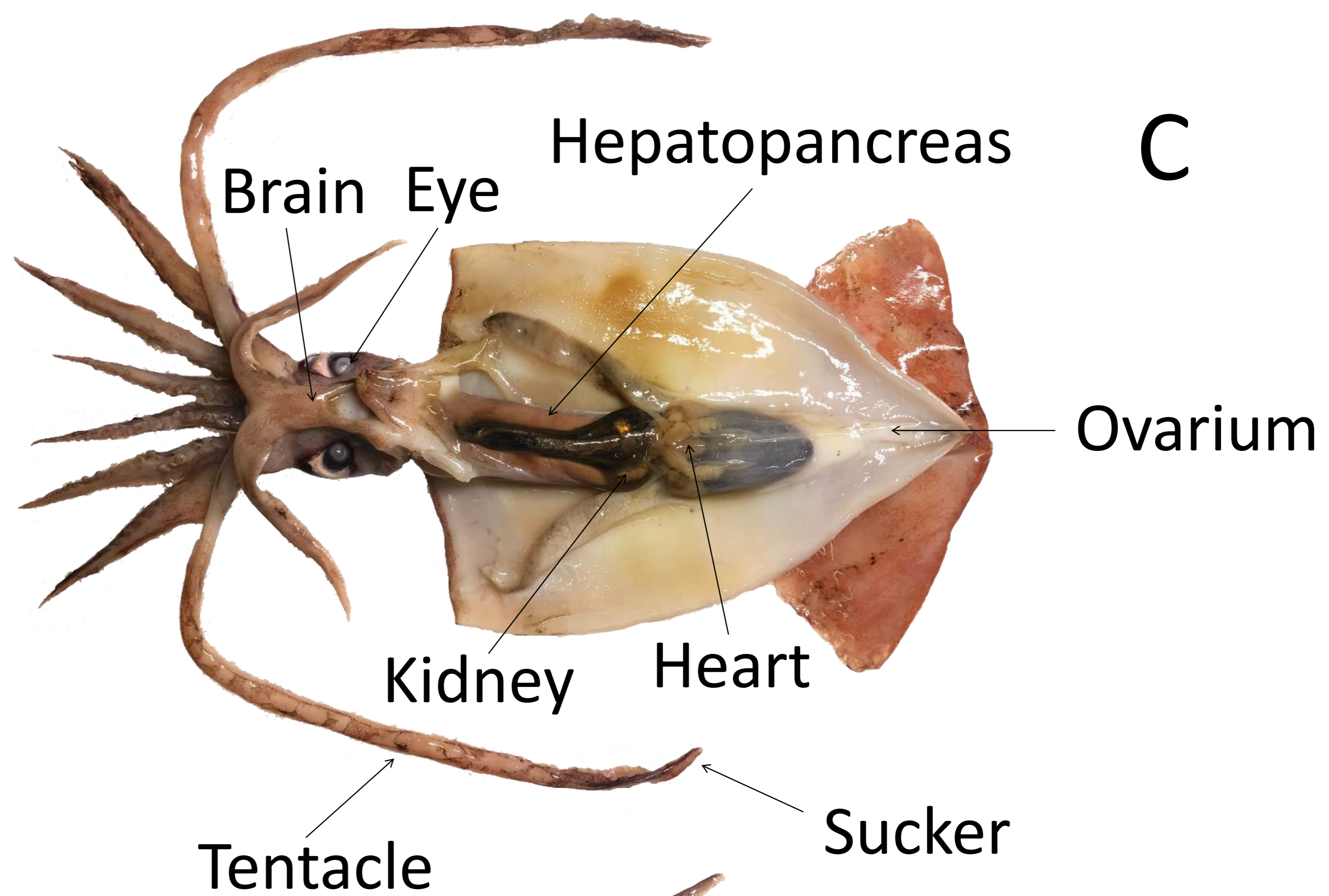

**D**

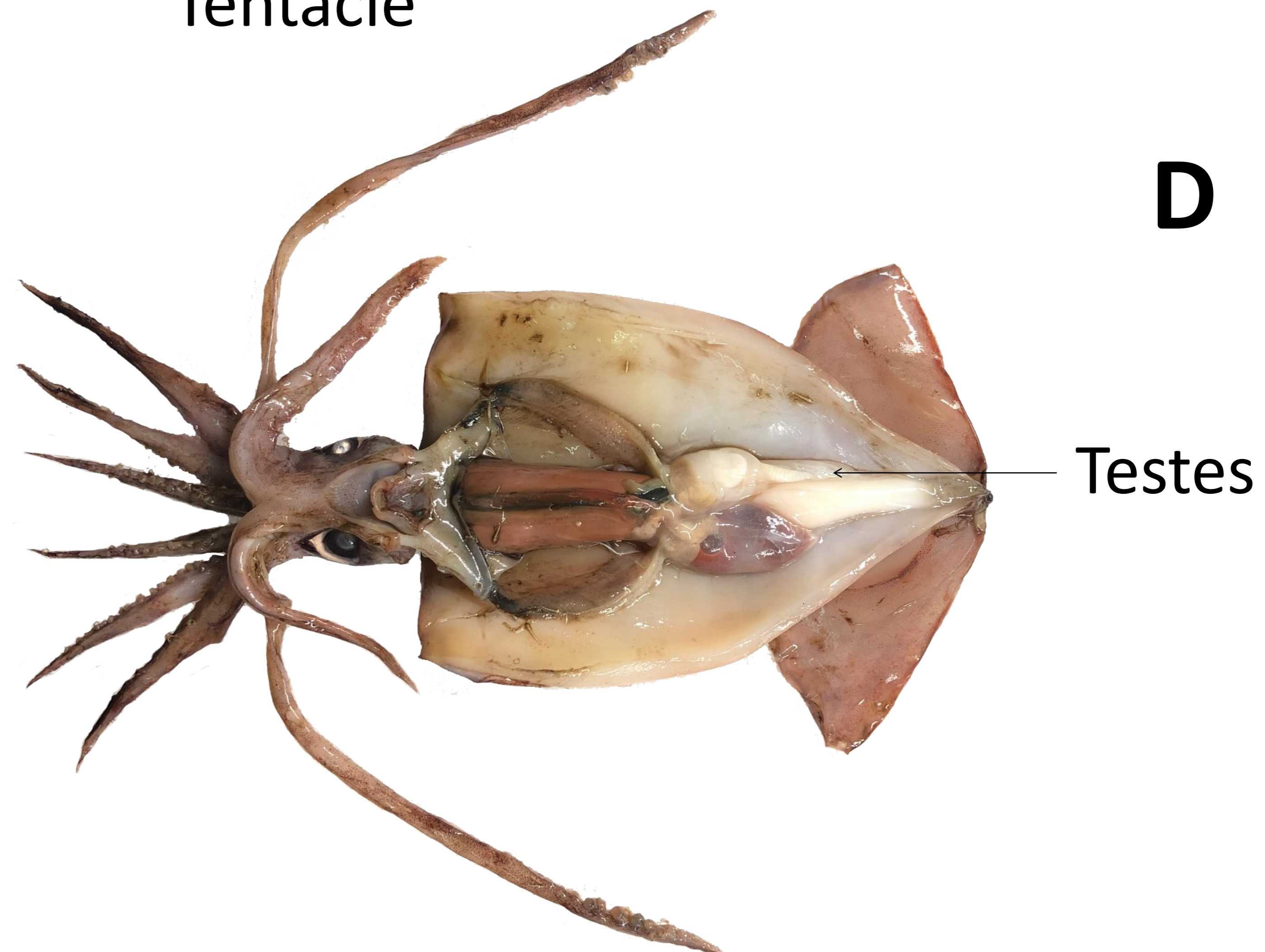

Supplement: Supplementary Figure S7 — Estimation of phylogeny and divergence times among purpleback flying squid species and their relatives The divergence time was indicated by the blue number near each node, while the 95% CI was indicated by the blue brackets and its specific value was colored in blue under the corresponding divergence time. Two softbound calibration time points had been applied: Octopus bimaculoides – Capitella teleta (585–679 MYA) and Octopus bimaculoides – Lottia gigantea (531–582 MYA). [file mmc1.pdf]

## Sequencing depth

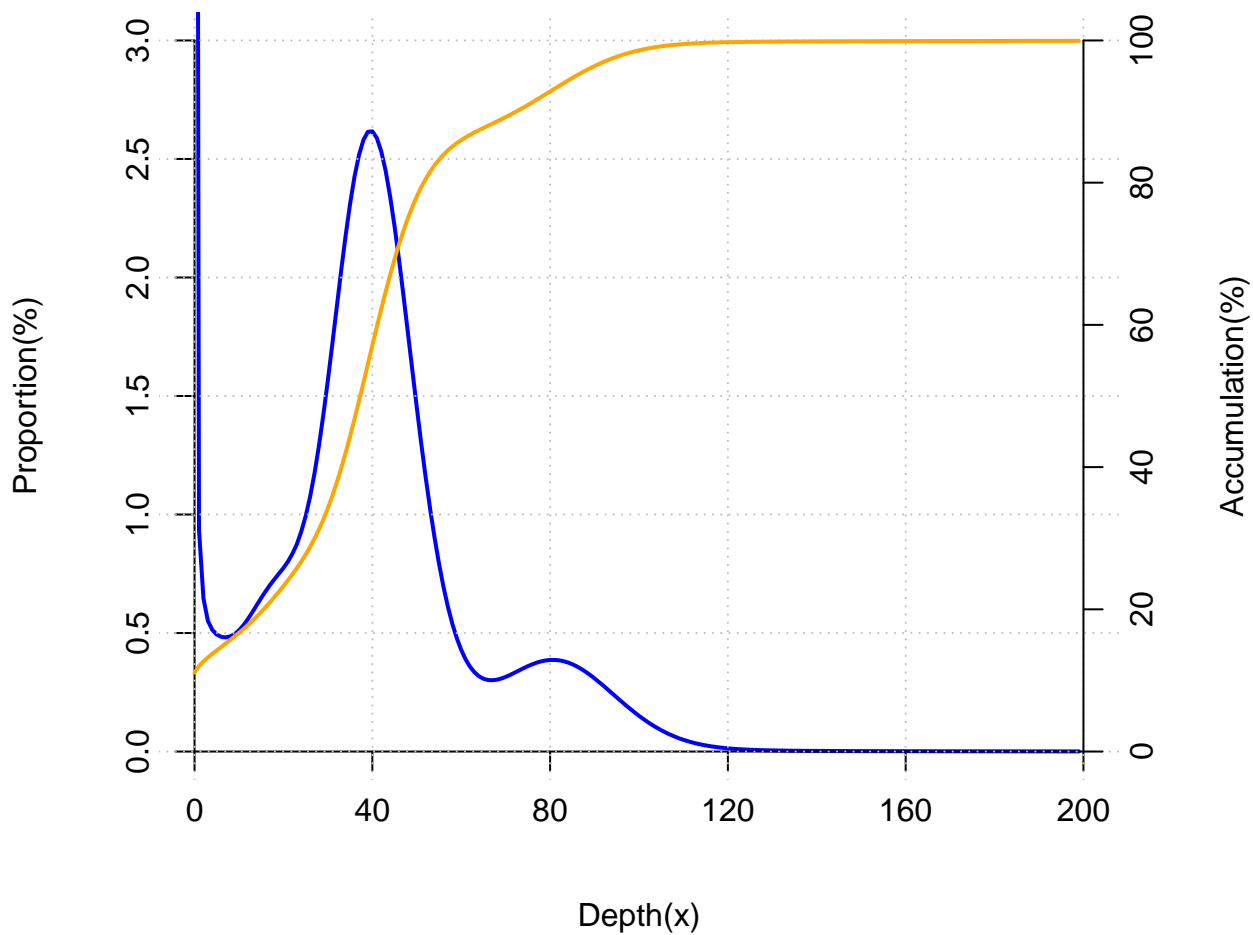

Supplement: Supplementary Figure S8 — Top 20 KEGG enriched pathway of the significantly expanded gene families for both Sthenoteuthis species [file mmc2.pdf]

# GenomeScope profile

len:2,988,857,525 bp uniq:23.7% het:2.77% kcov:23.5 err:0.109% dup:0.833% k:17

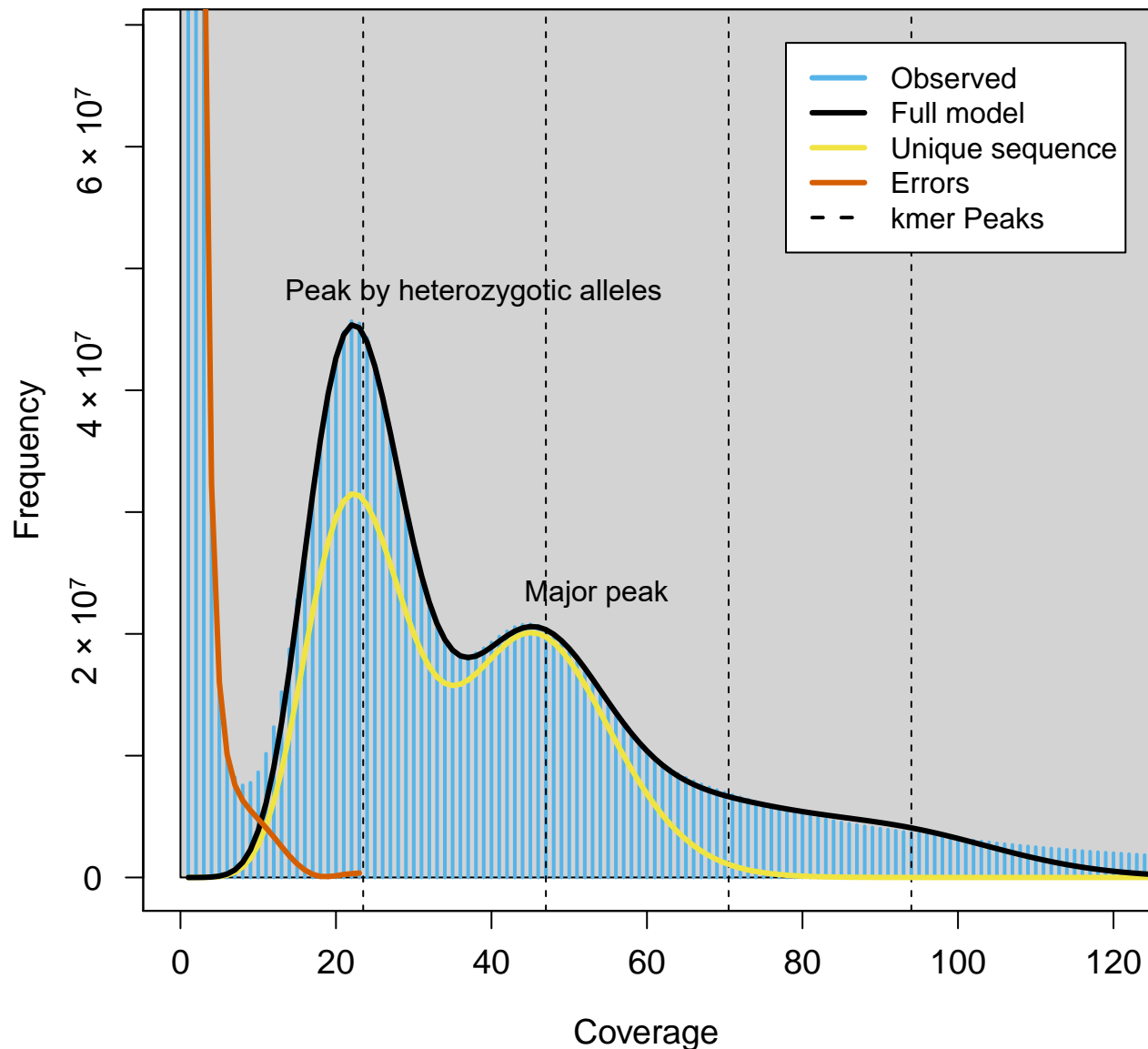

Supplement: Supplementary Figure S9 — The significantly enriched GO terms of the expanded gene families for both Sthenoteuthis species [file mmc3.pdf]

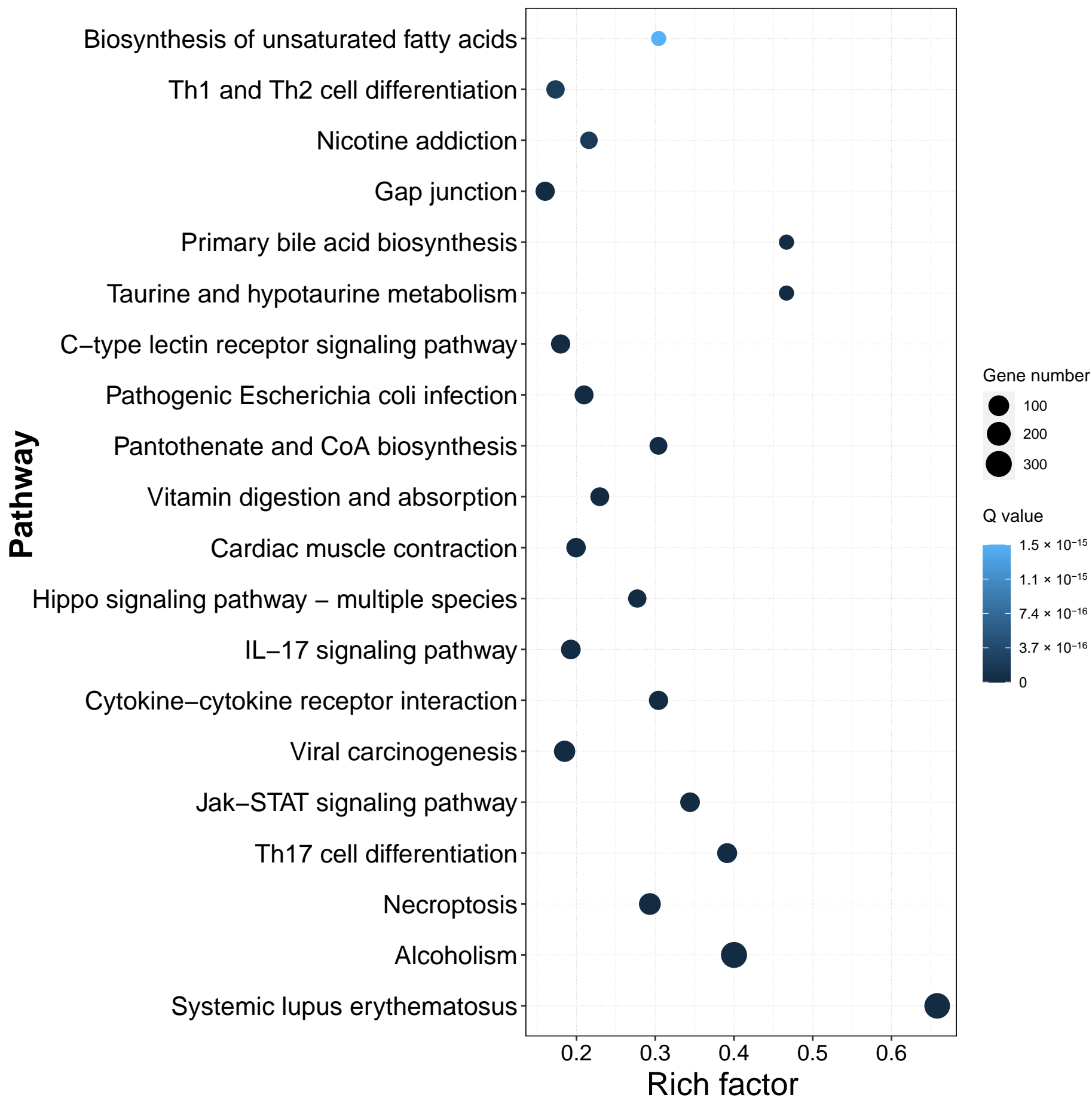

Supplement: Supplementary Figure S10 — Expression patterns of coexisting gene members of the SL20-1 subfamily in S. oualaniensis and Sthenoteuthis sp. Only those expressed gene copies were shown. Gene IDs and tissue information correspond to Figure 3C. Most of the significantly expanded genes were highly expressed in the eyes. [file mmc4.pdf]

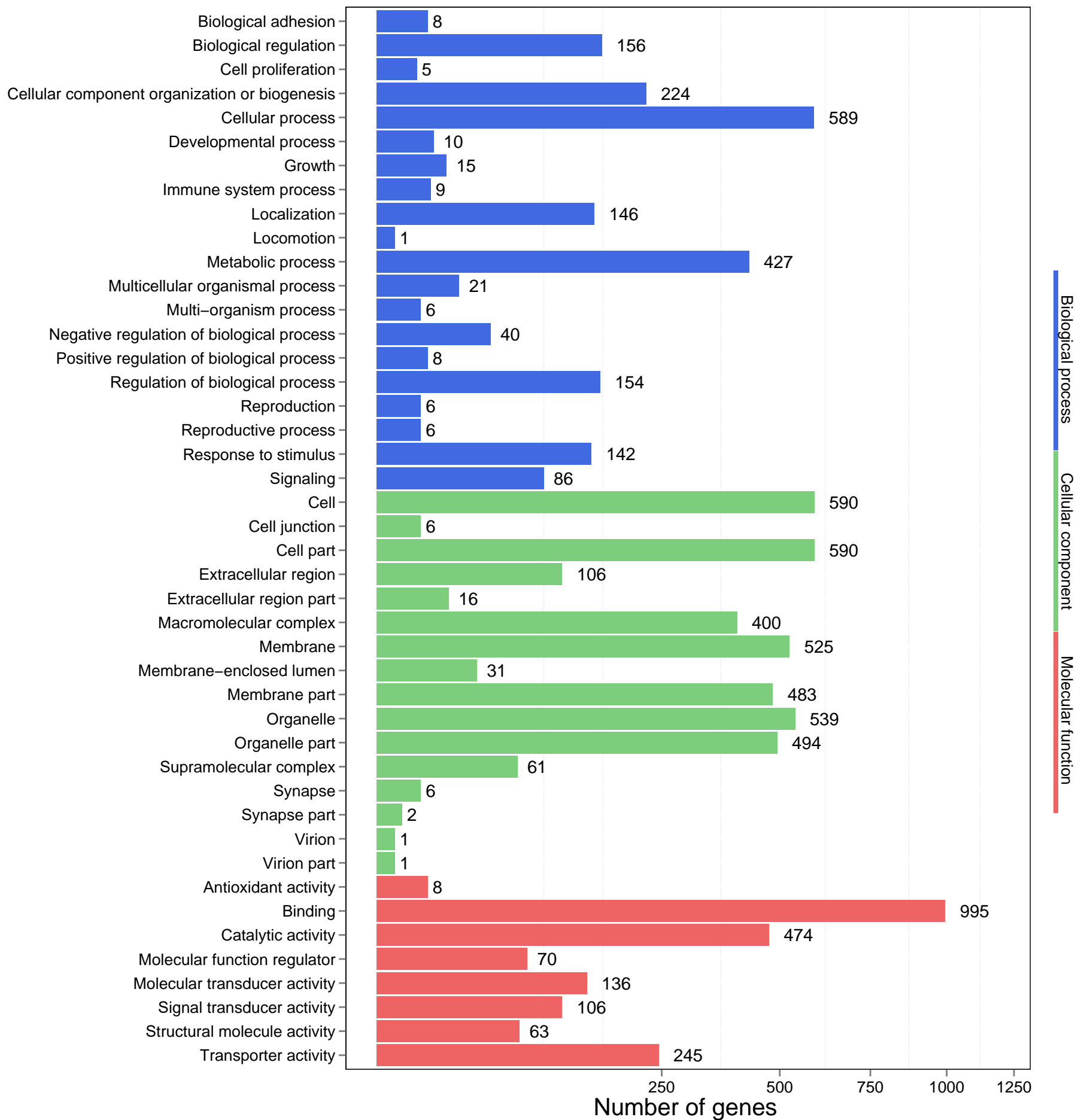

Supplement: Supplementary Figure S11 — Classification of the three major GO function annotations for the 66 PSGs of the two Sthenoteuthis lineage [file mmc5.pdf]

Gene ID

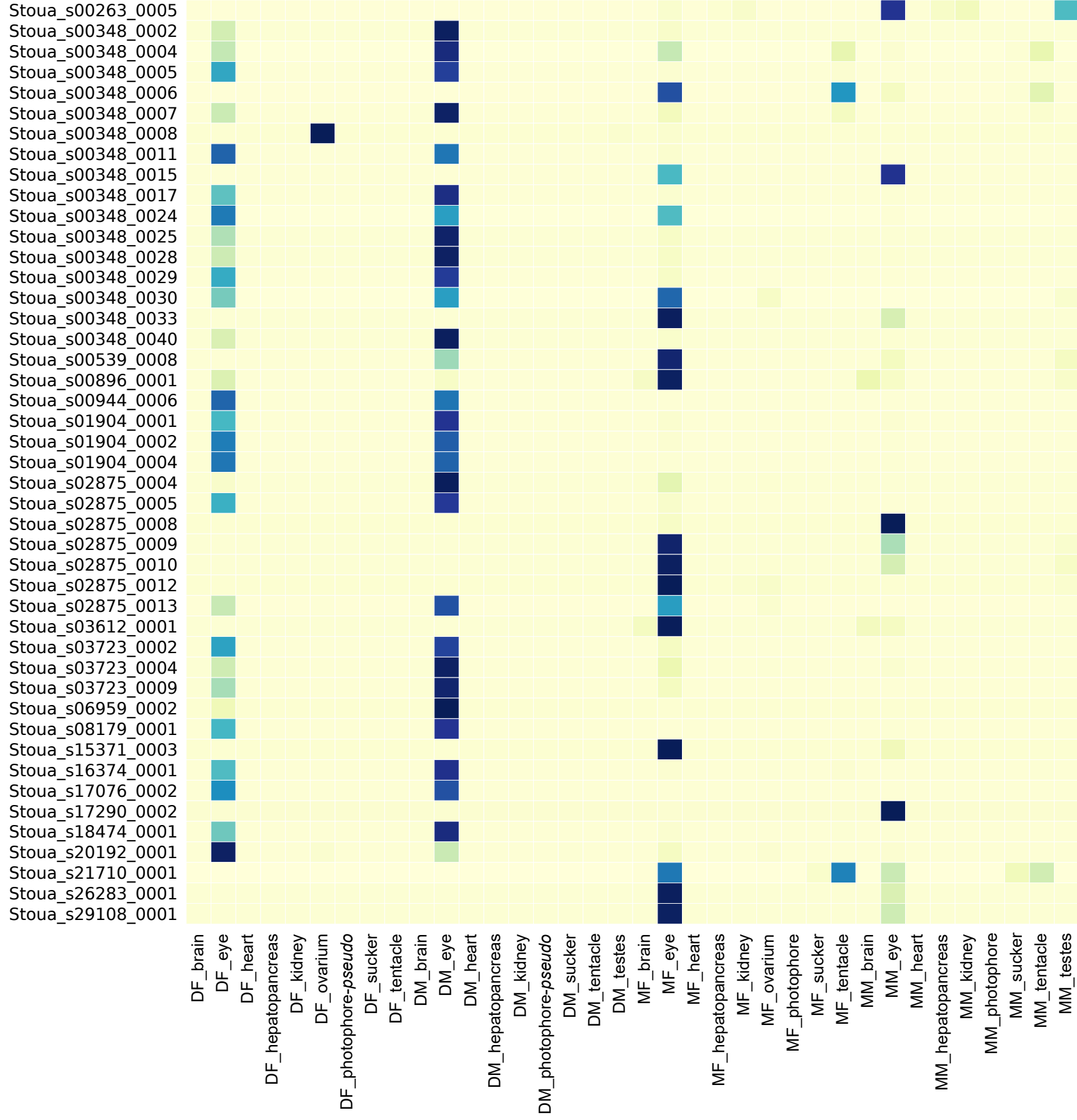

Quantile normalized RPKM

Supplement: Supplementary Figure S12 — The significantly enriched KEGG pathway of the positively selected genes for both Sthenoteuthis species [file mmc6.pdf]

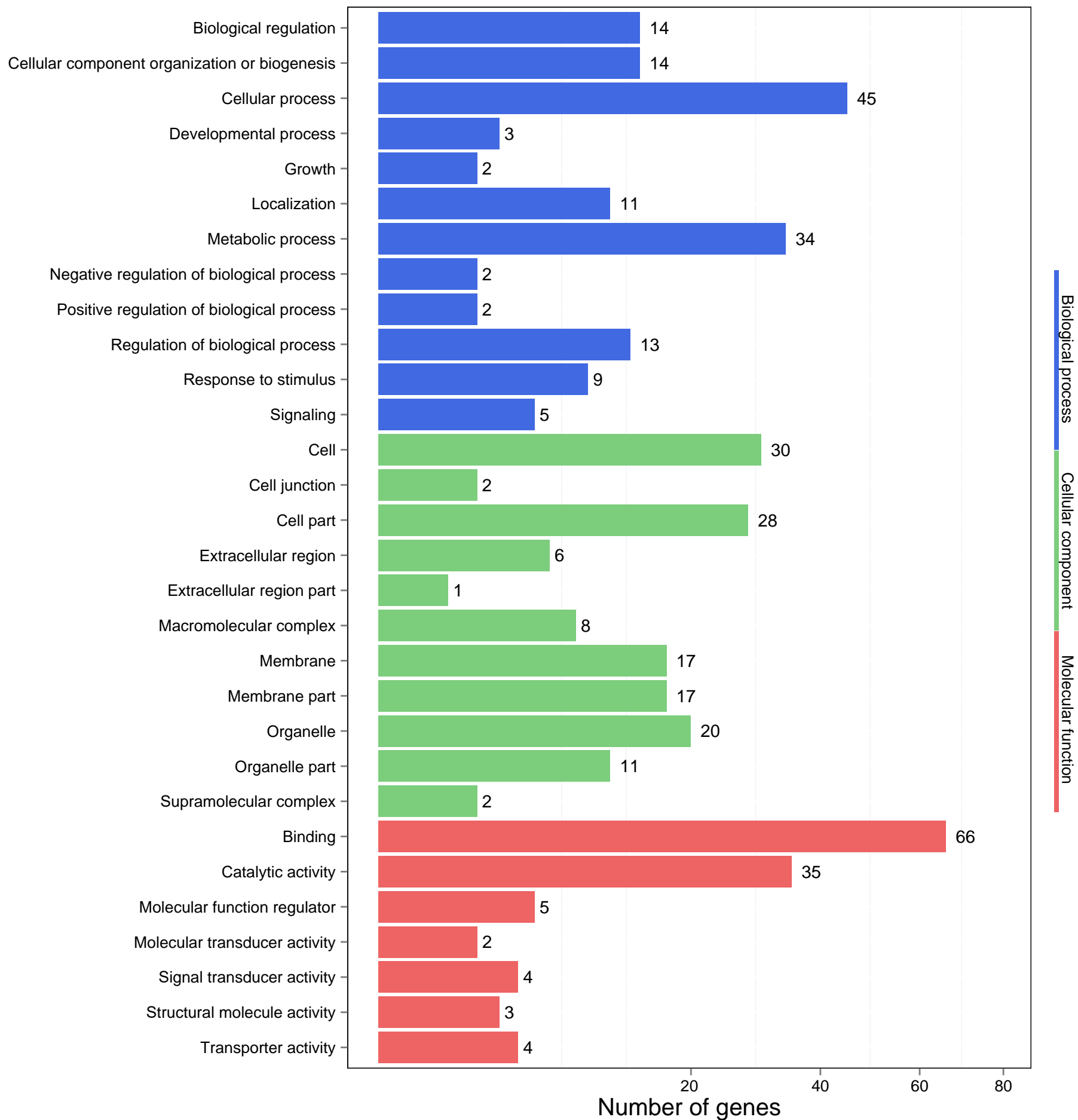

Supplement: Supplementary Figure S13 — Sequence alignments of IscS for the two Sthenoteuthis and other seven species from Mollusca and Annelida [file mmc7.pdf]

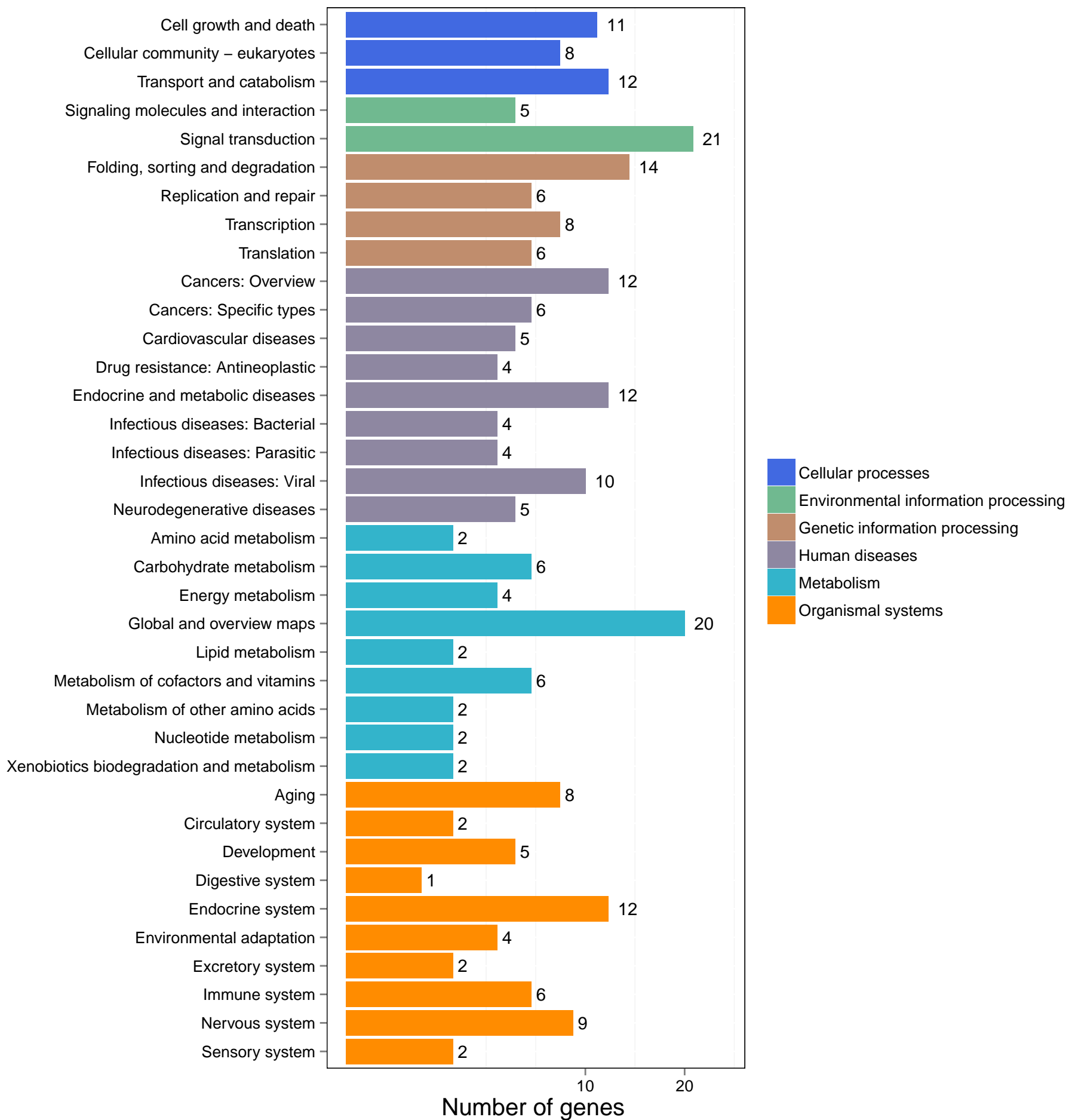

Supplement: Supplementary Figure S14 — Positive selection sites for genes PGK1 (Figure 4B) and IscS (Figure S13) shared by the two Sthenoteuthis were also detected from the giant squid (Architeuthis dux) [file mmc8.pdf]
