# Supplementary material for: Genomes of Two Flying Squid Species Provide Novel Insights into Adaptations of Cephalopods to Pelagic Life
Source: Genomics Proteomics Bioinformatics. 2022 Oct 7;20(6):1053–65. doi: 10.1016/j.gpb.2022.09.009 (PMC10225486; doi:10.1016/j.gpb.2022.09.009)
Supplement: Supplementary Table S1 [file mmc9.docx]

**Table S1**  **PacBio reads statistics of the two *Sthenoteuthis* species**

| **Library** | **Total bases (Gb)** | **Total reads number** | **Average length**  **(bp)** | **Max length (bp)** | **Min length (bp)** | **N50 length (bp)** |
| --- | --- | --- | --- | --- | --- | --- |
| r64048_20200304_014532-2_B01 | 196 | 14,755,426 | 13,305 | 263,237 | 50 | 16,488 |
| r64048_20200309_050911-4_D01 | 207 | 14,264,885 | 14,526 | 377,584 | 50 | 17,036 |

*Note*: Gb, gigabase; bp, base pair.
