# Supplementary material for: Genomes of Two Flying Squid Species Provide Novel Insights into Adaptations of Cephalopods to Pelagic Life
Source: Genomics Proteomics Bioinformatics. 2022 Oct 7;20(6):1053–65. doi: 10.1016/j.gpb.2022.09.009 (PMC10225486; doi:10.1016/j.gpb.2022.09.009)
Supplement: Supplementary Table S2 [file mmc10.docx]

**Table S2 Statistics of NGS sequencing**

| **Platform** | **Lane name** | **Reads count** | **Base (bp)** | **Length** | **Q20** | **Q30** | **GC (%)** |
| --- | --- | --- | --- | --- | --- | --- | --- |
| NGS | 171231_X602_FCH5GKMCCXY_L6_wHADPI064996-130 | 490,360,001 | 1.471E+11 | 150;150 | 96.72;93.78 | 92.33;86.93 | 34.63;34.91 |
|  | 171231_X602_FCH5GKMCCXY_L7_wHADPI064996-130 | 490,187,979 | 1.471E+11 | 150;150 | 96.72;93.60 | 92.40;86.58 | 34.60;34.87 |
|  | 171231_X602_FCH5GKMCCXY_L8_wHAIPI064995-133 | 490,049,960 | 1.47E+11 | 150;150 | 96.62;91.98 | 92.08;83.93 | 33.77;34.13 |
|  | 171231_X602_FCH5GHTCCXY_L4_WHSTHwwxDEAADWAAPEI-32 | 484,213,479 | 1.453E+11 | 150;150 | 97.95;94.72 | 95.23;89.63 | 36.20;36.39 |
|  | 171231_X602_FCH5GHTCCXY_L5_WHSTHwwxDEAADWAAPEI-32 | 484,008,671 | 1.452E+11 | 150;150 | 97.84;94.66 | 94.98;89.54 | 36.25;36.44 |
|  | 171231_X602_FCH5GHTCCXY_L1_WHSTHwwxDFAADLAAPEI-92 | 469,606,815 | 1.409E+11 | 150;150 | 97.64;94.31 | 94.50;88.77 | 35.94;36.21 |
|  | 171231_X602_FCH5GHTCCXY_L2_WHSTHwwxDFAADLAAPEI-92 | 468,872,994 | 1.407E+11 | 150;150 | 97.61;93.85 | 94.46;87.89 | 35.92;36.16 |
|  | 171231_X602_FCH5GHTCCXY_L3_WHSTHwwxDGAADTAAPEI-84 | 480,129,452 | 1.44E+11 | 150;150 | 97.72;94.81 | 94.61;89.43 | 35.52;35.72 |
|  | 171231_X602_FCH5GHTCCXY_L6_WHSTHwwxDHAADUAAPEI-86 | 480,589,371 | 1.442E+11 | 150;150 | 96.69;92.99 | 93.51;87.34 | 36.13;36.40 |
|  | 180106_I188_FCCBT7CANXX_L2_wHAMPI064997-131 | 239,118,372 | 5.978E+10 | 125;125 | 92.85;90.13 | 86.31;82.17 | 33.35;33.50 |
|  | 180106_I188_FCCBT7CANXX_L3_wHAMPI064997-131 | 239,565,426 | 5.989E+10 | 125;125 | 92.53;91.30 | 85.77;84.37 | 33.36;33.47 |
|  | 180106_I188_FCCBT7CANXX_L4_wHAMPI064997-131 | 239,654,642 | 5.991E+10 | 125;125 | 93.08;91.29 | 86.76;84.39 | 33.36;33.48 |
|  | 180106_I188_FCCBT7CANXX_L5_wHAMPI064997-131 | 240,438,866 | 6.011E+10 | 125;125 | 92.86;87.26 | 86.37;77.71 | 33.36;33.68 |
|  | 171212_X603_FCH5GL3CCXY_L1_wHADPI064996-130 | 485,174,438 | 1.456E+11 | 150;150 | 97.83;96.22 | 94.67;90.86 | 34.37;34.52 |
|  | 171226_X603_FCH5GL3CCXY_L1_wHADPI064996-130 | 497,996,920 | 1.494E+11 | 150;150 | 97.37;95.07 | 93.97;89.35 | 34.54;34.78 |
|  | 171212_X603_FCH5GL3CCXY_L2_wHAIPI064995-133 | 477,806,525 | 1.433E+11 | 150;150 | 98.04;94.25 | 95.09;87.36 | 33.67;33.88 |
|  | 171226_X603_FCH5GL3CCXY_L2_wHAIPI064995-133 | 511,818,498 | 1.535E+11 | 150;150 | 97.15;91.34 | 93.59;83.54 | 33.82;34.31 |
|  | 171224_X603_FCH5GJ2CCXY_L6_WHSTHwwxDEAADWAAPEI-32 | 496,803,120 | 1.49E+11 | 150;150 | 97.76;94.21 | 94.83;88.54 | 36.26;36.39 |
|  | 171224_X603_FCH5GJ2CCXY_L2_WHSTHwwxDFAADLAAPEI-92 | 490,644,651 | 1.472E+11 | 150;150 | 97.56;93.51 | 94.47;87.41 | 36.04;36.24 |
|  | 171224_X603_FCH5GJ2CCXY_L3_WHSTHwwxDGAADTAAPEI-84 | 493,234,764 | 1.48E+11 | 150;150 | 97.51;94.07 | 94.30;87.99 | 35.61;35.74 |
|  | 171224_X603_FCH5GJ2CCXY_L7_WHSTHwwxDHAADUAAPEI-86 | 490,555,492 | 1.472E+11 | 150;150 | 96.54;92.13 | 93.18;85.62 | 36.07;36.21 |
|  | 171224_X603_FCH5GJ2CCXY_L4_WHSTHwwxDIAADVAAPEI-88 | 472,614,848 | 1.418E+11 | 150;150 | 96.66;92.94 | 93.15;86.79 | 34.83;35.18 |

*Note*: NGS, Next Generation Sequencing.
