# Supplementary material for: Genomes of Two Flying Squid Species Provide Novel Insights into Adaptations of Cephalopods to Pelagic Life
Source: Genomics Proteomics Bioinformatics. 2022 Oct 7;20(6):1053–65. doi: 10.1016/j.gpb.2022.09.009 (PMC10225486; doi:10.1016/j.gpb.2022.09.009)
Supplement: Supplementary Table S3 [file mmc11.docx]

**Table S3**  **Statistics of 10× sequencing**

| **Platform** | **Lane name** | **Reads count** | **Base (bp)** | **Length** | **Q20** | **Q30** | **GC** |
| --- | --- | --- | --- | --- | --- | --- | --- |
| 10× | 180407_X495_FCHKG7KCCXY_L7_STHwwxD-AD-G10-1 | 41,845,197,900 | 4.18E+10 | 150;150 | 96.83;92.72 | 92.70;85.77 | 36.79;34.90 |
|  | 180407_X495_FCHKG7KCCXY_L7_STHwwxD-AD-G10-2 | 29,492,050,800 | 2.95E+10 | 150;150 | 96.94;93.38 | 92.96;87.08 | 36.73;34.77 |
|  | 180407_X495_FCHKG7KCCXY_L7_STHwwxD-AD-G10-3 | 25,025,630,400 | 2.5E+10 | 150;150 | 96.87;93.14 | 92.81;86.62 | 36.77;34.84 |
|  | 180407_X495_FCHKG7KCCXY_L7_STHwwxD-AD-G10-4 | 29,713,203,600 | 2.97E+10 | 150;150 | 96.87;93.21 | 92.82;86.74 | 36.76;34.82 |
|  | 180407_X495_FCHKGNNCCXY_L6_STHwwxD-AD-G10-1 | 42,188,685,300 | 4.22E+10 | 150;150 | 97.32;94.40 | 93.79;88.80 | 36.79;34.83 |
|  | 180407_X495_FCHKGNNCCXY_L6_STHwwxD-AD-G10-2 | 29,634,741,900 | 2.96E+10 | 150;150 | 97.45;95.01 | 94.10;90.05 | 36.73;34.72 |
|  | 180407_X495_FCHKGNNCCXY_L6_STHwwxD-AD-G10-3 | 25114150500 | 2.51E+10 | 150;150 | 97.35;94.78 | 93.89;89.61 | 36.76;34.77 |
|  | 180407_X495_FCHKGNNCCXY_L6_STHwwxD-AD-G10-4 | 29,838,146,700 | 2.98E+10 | 150;150 | 97.37;94.86 | 93.91;89.73 | 36.75;34.75 |
|  | 180407_X495_FCHKGNNCCXY_L7_STHwwxD-AD-G10-1 | 41,858,298,000 | 4.19E+10 | 150;150 | 97.35;94.27 | 93.85;88.57 | 36.79;34.85 |
|  | 180407_X495_FCHKGNNCCXY_L7_STHwwxD-AD-G10-2 | 29,377,740,600 | 2.94E+10 | 150;150 | 97.48;94.90 | 94.15;89.84 | 36.72;34.73 |
|  | 180407_X495_FCHKGNNCCXY_L7_STHwwxD-AD-G10-3 | 24,917,654,100 | 2.49E+10 | 150;150 | 97.38;94.67 | 93.94;89.40 | 36.76;34.78 |
|  | 180407_X495_FCHKGNNCCXY_L7_STHwwxD-AD-G10-4 | 29,626,963,800 | 2.96E+10 | 150;150 | 97.39;94.74 | 93.97;89.52 | 36.75;34.77 |
|  | 180407_X528_FCHKGMVCCXY_L3_STHwwxD-AD-G10-1 | 42,281,920,200 | 4.23E+10 | 150;150 | 97.10;93.67 | 93.35;87.83 | 36.84;34.95 |
|  | 180407_X528_FCHKGMVCCXY_L3_STHwwxD-AD-G10-2 | 29,725,274,100 | 2.97E+10 | 150;150 | 97.21;94.21 | 93.61;88.91 | 36.78;34.83 |
|  | 180407_X528_FCHKGMVCCXY_L3_STHwwxD-AD-G10-3 | 25165895400 | 2.52E+10 | 150;150 | 97.13;93.99 | 93.45;88.49 | 36.81;34.90 |
|  | 180407_X528_FCHKGMVCCXY_L3_STHwwxD-AD-G10-4 | 29,904,066,600 | 2.99E+10 | 150;150 | 97.14;94.06 | 93.47;88.60 | 36.80;34.88 |
|  | 180407_X528_FCHKGMVCCXY_L4_STHwwxD-AD-G10-1 | 42,916,878,600 | 4.29E+10 | 150;150 | 97.21;93.91 | 93.61;88.35 | 36.84;34.96 |
|  | 180407_X528_FCHKGMVCCXY_L4_STHwwxD-AD-G10-2 | 30,144,276,300 | 3.01E+10 | 150;150 | 97.31;94.43 | 93.86;89.39 | 36.78;34.84 |
|  | 180407_X528_FCHKGMVCCXY_L4_STHwwxD-AD-G10-3 | 25,489,609,800 | 2.55E+10 | 150;150 | 97.23;94.22 | 93.70;88.99 | 36.82;34.91 |
|  | 180407_X528_FCHKGMVCCXY_L4_STHwwxD-AD-G10-4 | 30305369700 | 3.03E+10 | 150;150 | 97.25;94.30 | 93.73;89.12 | 36.81;34.88 |
|  | 180407_X528_FCHKGMVCCXY_L5_STHwwxD-AD-G10-1 | 43,003,608,900 | 4.3E+10 | 150;150 | 97.23;94.01 | 93.67;88.48 | 36.83;34.94 |
|  | 180407_X528_FCHKGMVCCXY_L5_STHwwxD-AD-G10-2 | 30,197,941,200 | 3.02E+10 | 150;150 | 97.33;94.54 | 93.91;89.54 | 36.77;34.82 |
|  | 180407_X528_FCHKGMVCCXY_L5_STHwwxD-AD-G10-3 | 25,552,044,900 | 2.56E+10 | 150;150 | 97.25;94.33 | 93.75;89.15 | 36.81;34.88 |
|  | 180407_X528_FCHKGMVCCXY_L5_STHwwxD-AD-G10-4 | 30,391,401,000 | 3.04E+10 | 150;150 | 97.27;94.41 | 93.78;89.29 | 36.80;34.86 |
