# Supplementary material for: Genomes of Two Flying Squid Species Provide Novel Insights into Adaptations of Cephalopods to Pelagic Life
Source: Genomics Proteomics Bioinformatics. 2022 Oct 7;20(6):1053–65. doi: 10.1016/j.gpb.2022.09.009 (PMC10225486; doi:10.1016/j.gpb.2022.09.009)
Supplement: Supplementary Table S4 [file mmc12.docx]

**Table S4 Statistics of Hi-C sequencing**

| **Platform** | **Lane name** | **Reads** | **Base** | **Length** | **Q20** | **Q30** | **GC** |
| --- | --- | --- | --- | --- | --- | --- | --- |
| Hi-C | 190323_I25_V300014809_L1_WHRDSTHwwxDAAAMAB-509 | 51,681,097 | 15,504,329,100 | 150;150 | 96.23;94.65 | 87.93;84.58 | 35.86;36.01 |
|  | 190323_I25_V300014809_L1_WHRDSTHwwxDAAAMAB-510 | 60,349,538 | 18,104,861,400 | 150;150 | 96.14;94.42 | 87.71;84.16 | 35.87;36.01 |
|  | 190323_I25_V300014809_L1_WHRDSTHwwxDAAAMAB-511 | 64,110,433 | 19,233,129,900 | 150;150 | 96.23;94.89 | 87.98;85.19 | 35.86;35.99 |
|  | 190323_I25_V300014809_L1_WHRDSTHwwxDAAAMAB-512 | 59,664,912 | 17,899,473,600 | 150;150 | 96.32;95.24 | 88.24;86.00 | 35.87;35.96 |
|  | 190323_I25_V300014809_L1_WHRDSTHwwxDAAAMAB-513 | 47,233,033 | 14,169,909,900 | 150;150 | 96.30;94.97 | 88.19;85.47 | 35.82;35.96 |
|  | 190323_I25_V300014809_L1_WHRDSTHwwxDAAAMAB-514 | 54,825,110 | 16,447,533,000 | 150;150 | 96.05;95.06 | 87.56;85.56 | 35.89;35.98 |
|  | 190323_I25_V300014809_L1_WHRDSTHwwxDAAAMAB-515 | 52,303,566 | 15,691,069,800 | 150;150 | 96.19;94.69 | 87.83;84.74 | 35.86;36.02 |
|  | 190323_I25_V300014809_L1_WHRDSTHwwxDAAAMAB-516 | 66,507,454 | 19,952,236,200 | 150;150 | 96.17;94.68 | 87.80;84.70 | 35.91;36.02 |
|  | 190113_I412_CL100111910_L1_WHRDSTHwwxDAAAMAB-509 | 62,028,564 | 12,405,712,800 | 100;100 | 97.49;92.99 | 90.16;80.48 | 35.57;35.56 |
|  | 190113_I412_CL100111910_L1_WHRDSTHwwxDAAAMAB-510 | 74,317,337 | 14,863,467,400 | 100;100 | 97.53;93.17 | 90.26;80.87 | 35.59;35.52 |
|  | 190113_I412_CL100111910_L1_WHRDSTHwwxDAAAMAB-511 | 78071192 | 15,614,238,400 | 100;100 | 97.55;93.33 | 90.32;81.24 | 35.58;35.54 |
|  | 190113_I412_CL100111910_L1_WHRDSTHwwxDAAAMAB-512 | 74,175,906 | 14,835,181,200 | 100;100 | 97.58;93.34 | 90.43;81.26 | 35.58;35.53 |
|  | 190113_I412_CL100111910_L1_WHRDSTHwwxDAAAMAB-513 | 57,232,743 | 11,446,548,600 | 100;100 | 97.52;93.17 | 90.24;80.93 | 35.54;35.54 |
|  | 190113_I412_CL100111910_L1_WHRDSTHwwxDAAAMAB-514 | 67,704,437 | 13,540,887,400 | 100;100 | 97.40;93.16 | 89.93;80.89 | 35.60;35.56 |
|  | 190113_I412_CL100111910_L1_WHRDSTHwwxDAAAMAB-515 | 64,249,139 | 12,849,827,800 | 100;100 | 97.57;93.45 | 90.38;81.52 | 35.57;35.55 |
|  | 190113_I412_CL100111910_L1_WHRDSTHwwxDAAAMAB-516 | 81,973,766 | 16,394,753,200 | 100;100 | 97.45;93.25 | 90.06;81.05 | 35.62;35.53 |
|  | 190312_I525_CL100110998_L1_WHRDSTHwwxDAAAMAB-509 | 73,023,423 | 14,604,684,600 | 100;100 | 96.76;91.59 | 88.76;77.58 | 35.63;35.49 |
|  | 190312_I525_CL100110998_L1_WHRDSTHwwxDAAAMAB-510 | 87,849,948 | 17,569,989,600 | 100;100 | 96.62;91.60 | 88.40;77.65 | 35.68;35.50 |
|  | 190312_I525_CL100110998_L1_WHRDSTHwwxDAAAMAB-511 | 94,088,826 | 18,817,765,200 | 100;100 | 96.85;91.98 | 89.02;78.44 | 35.63;35.49 |
|  | 190312_I525_CL100110998_L1_WHRDSTHwwxDAAAMAB-512 | 89,867,469 | 17,973,493,800 | 100;100 | 96.82;92.15 | 88.94;78.78 | 35.66;35.50 |
|  | 190312_I525_CL100110998_L1_WHRDSTHwwxDAAAMAB-513 | 68,964,948 | 13,792,989,600 | 100;100 | 96.86;91.66 | 89.12;77.83 | 35.59;35.47 |
|  | 190312_I525_CL100110998_L1_WHRDSTHwwxDAAAMAB-514 | 78,554,916 | 15,710,983,200 | 100;100 | 95.70;91.63 | 86.04;77.68 | 35.74;35.52 |
|  | 190312_I525_CL100110998_L1_WHRDSTHwwxDAAAMAB-515 | 77,209,126 | 15,441,825,200 | 100;100 | 96.84;92.09 | 88.97;78.69 | 35.64;35.53 |
|  | 190312_I525_CL100110998_L1_WHRDSTHwwxDAAAMAB-516 | 97,383,520 | 19,476,704,000 | 100;100 | 96.21;92.07 | 87.31;78.58 | 35.73;35.49 |
|  | 190312_I525_CL100110998_L2_WHRDSTHwwxDAAAMAB-509 | 79,137,857 | 15,827,571,400 | 100;100 | 97.31;92.37 | 89.56;79.06 | 35.58;35.50 |
|  | 190312_I525_CL100110998_L2_WHRDSTHwwxDAAAMAB-510 | 93,097,243 | 18,619,448,600 | 100;100 | 97.27;92.43 | 89.46;79.23 | 35.62;35.48 |
|  | 190312_I525_CL100110998_L2_WHRDSTHwwxDAAAMAB-511 | 101,327,211 | 20,265,442,200 | 100;100 | 97.40;92.73 | 89.86;79.91 | 35.59;35.48 |
|  | 190312_I525_CL100110998_L2_WHRDSTHwwxDAAAMAB-512 | 95,234,920 | 19,046,984,000 | 100;100 | 97.39;92.80 | 89.81;80.03 | 35.61;35.49 |
|  | 190312_I525_CL100110998_L2_WHRDSTHwwxDAAAMAB-513 | 73,296,020 | 14,659,204,000 | 100;100 | 97.37;92.40 | 89.75;79.16 | 35.55;35.48 |
|  | 190312_I525_CL100110998_L2_WHRDSTHwwxDAAAMAB-514 | 84,710,041 | 16,942,008,200 | 100;100 | 96.68;92.65 | 87.90;79.69 | 35.64;35.51 |
|  | 190312_I525_CL100110998_L2_WHRDSTHwwxDAAAMAB-515 | 82,677,852 | 16,535,570,400 | 100;100 | 97.40;92.86 | 89.85;80.20 | 35.59;35.50 |
|  | 190312_I525_CL100110998_L2_WHRDSTHwwxDAAAMAB-516 | 104,695,019 | 20,939,003,800 | 100;100 | 96.99;92.82 | 88.72;80.06 | 35.66;35.48 |
|  | 190312_I569_CL100112082_L1_WHRDSTHwwxDAAAMAB-509 | 75,811,112 | 15,162,222,400 | 100;100 | 97.53;93.13 | 89.76;80.68 | 35.58;35.56 |
|  | 190312_I569_CL100112082_L1_WHRDSTHwwxDAAAMAB-510 | 89,608,806 | 17,921,761,200 | 100;100 | 97.51;93.14 | 89.70;80.78 | 35.61;35.54 |
|  | 190312_I569_CL100112082_L1_WHRDSTHwwxDAAAMAB-511 | 96,092,960 | 19,218,592,000 | 100;100 | 97.57;93.43 | 89.90;81.38 | 35.60;35.55 |
|  | 190312_I569_CL100112082_L1_WHRDSTHwwxDAAAMAB-512 | 90,603,751 | 18,120,750,200 | 100;100 | 97.59;93.49 | 89.95;81.49 | 35.61;35.55 |
|  | 190312_I569_CL100112082_L1_WHRDSTHwwxDAAAMAB-513 | 70,591,166 | 14,118,233,200 | 100;100 | 97.53;93.20 | 89.78;80.93 | 35.55;35.55 |
|  | 190312_I569_CL100112082_L1_WHRDSTHwwxDAAAMAB-514 | 81,589,261 | 16,317,852,200 | 100;100 | 97.38;93.21 | 89.36;80.89 | 35.62;35.58 |
|  | 190312_I569_CL100112082_L1_WHRDSTHwwxDAAAMAB-515 | 78,397,566 | 15,679,513,200 | 100;100 | 97.59;93.54 | 89.96;81.63 | 35.59;35.57 |
|  | 190312_I569_CL100112082_L1_WHRDSTHwwxDAAAMAB-516 | 99,394,009 | 19,878,801,800 | 100;100 | 97.42;93.44 | 89.47;81.39 | 35.65;35.55 |
|  | 190312_I569_CL100112082_L2_WHRDSTHwwxDAAAMAB-509 | 77,644,100 | 15,528,820,000 | 100;100 | 97.49;93.36 | 89.83;80.86 | 35.60;35.53 |
|  | 190312_I569_CL100112082_L2_WHRDSTHwwxDAAAMAB-510 | 90,986,991 | 18,197,398,200 | 100;100 | 97.49;93.39 | 89.83;80.98 | 35.63;35.51 |
|  | 190312_I569_CL100112082_L2_WHRDSTHwwxDAAAMAB-511 | 97,773,990 | 19,554,798,000 | 100;100 | 97.53;93.64 | 89.97;81.54 | 35.62;35.52 |
|  | 190312_I569_CL100112082_L2_WHRDSTHwwxDAAAMAB-512 | 91,650,661 | 18,330,132,200 | 100;100 | 97.55;93.69 | 90.01;81.62 | 35.63;35.52 |
|  | 190312_I569_CL100112082_L2_WHRDSTHwwxDAAAMAB-513 | 72,167,629 | 14,433,525,800 | 100;100 | 97.50;93.47 | 89.87;81.16 | 35.57;35.52 |
|  | 190312_I569_CL100112082_L2_WHRDSTHwwxDAAAMAB-514 | 82,894,530 | 16,578,906,000 | 100;100 | 97.35;93.44 | 89.45;81.08 | 35.64;35.55 |
|  | 190312_I569_CL100112082_L2_WHRDSTHwwxDAAAMAB-515 | 79,691,338 | 15,938,267,600 | 100;100 | 97.56;93.74 | 90.05;81.78 | 35.61;35.54 |
|  | 190312_I569_CL100112082_L2_WHRDSTHwwxDAAAMAB-516 | 101,040,813 | 20,208,162,600 | 100;100 | 97.40;93.64 | 89.59;81.51 | 35.67;35.53 |
|  | 190313_I101_CL100110996_L1_WHRDSTHwwxDAAAMAB-509 | 71,973,464 | 14,394,692,800 | 100;100 | 96.87;92.91 | 88.22;79.14 | 35.59;35.51 |
|  | 190313_I101_CL100110996_L1_WHRDSTHwwxDAAAMAB-510 | 84,325,203 | 16,865,040,600 | 100;100 | 96.82;92.76 | 88.07;78.92 | 35.61;35.50 |
|  | 190313_I101_CL100110996_L1_WHRDSTHwwxDAAAMAB-511 | 91,855,713 | 18,371,142,600 | 100;100 | 96.90;93.18 | 88.37;79.82 | 35.60;35.50 |
|  | 190313_I101_CL100110996_L1_WHRDSTHwwxDAAAMAB-512 | 85,194,550 | 17,038,910,000 | 100;100 | 96.96;93.34 | 88.50;80.08 | 35.61;35.51 |
|  | 190313_I101_CL100110996_L1_WHRDSTHwwxDAAAMAB-513 | 65,864,264 | 13,172,852,800 | 100;100 | 96.86;92.80 | 88.26;79.01 | 35.56;35.50 |
|  | 190313_I101_CL100110996_L1_WHRDSTHwwxDAAAMAB-514 | 77,782,321 | 15,556,464,200 | 100;100 | 96.76;92.99 | 87.98;79.37 | 35.63;35.54 |
|  | 190313_I101_CL100110996_L1_WHRDSTHwwxDAAAMAB-515 | 75,250,064 | 15,050,012,800 | 100;100 | 96.92;93.28 | 88.39;80.09 | 35.59;35.51 |
|  | 190313_I101_CL100110996_L1_WHRDSTHwwxDAAAMAB-516 | 96,708,875 | 19,341,775,000 | 100;100 | 96.79;93.19 | 88.02;79.84 | 35.65;35.50 |
