# Supplementary material for: Genomes of Two Flying Squid Species Provide Novel Insights into Adaptations of Cephalopods to Pelagic Life
Source: Genomics Proteomics Bioinformatics. 2022 Oct 7;20(6):1053–65. doi: 10.1016/j.gpb.2022.09.009 (PMC10225486; doi:10.1016/j.gpb.2022.09.009)
Supplement: Supplementary Table S5 [file mmc13.docx]

**Table S5 Statistics of the predicted gene sets for two *Sthenoteuthis* species and the comparison with other relatives**

| **Species** | **Average mRNA length** | **Average CDS length** | **Average exon length** | **Average exon number** | **Average intron length** | **Gene number** | **GC content** |
| --- | --- | --- | --- | --- | --- | --- | --- |
| *Octopus bimaculoides* | 16,136.23 | 906.34 | 207.69 | 4.36 | 3840.51 | 26,936 | 29.90% |
| *Euprymna scolopes* | 18,007.25 | 864.75 | 232.14 | 3.73 | 6290.36 | 33,793 | 21.80% |
| *Octopus vulgaris* | 71,229.43 | 1815.68 | 186.36 | 9.74 | 6569.61 | 25,643 | 29.90% |
| *Sthenoteuthis oualaniensis* | 26,772.23 | 1174.35 | 201.84 | 5.82 | 4603.80 | 26,646 | 33.07% |
| *Sthenoteuthis* sp*.* | 36,141.98 | 1250.93 | 180.68 | 6.92 | 4878.78 | 28,715 | 33.00% |
