# Supplementary material for: Genomes of Two Flying Squid Species Provide Novel Insights into Adaptations of Cephalopods to Pelagic Life
Source: Genomics Proteomics Bioinformatics. 2022 Oct 7;20(6):1053–65. doi: 10.1016/j.gpb.2022.09.009 (PMC10225486; doi:10.1016/j.gpb.2022.09.009)
Supplement: Supplementary Table S6 [file mmc14.docx]

**Table S6 The sequencing reads information used for transcriptomes assembly from nine tissues**

| **Species** | **Sample** | **Reads counts** | **Average**  **length** | **Min length** | **Max length** |
| --- | --- | --- | --- | --- | --- |
| *S. oualaniensis*  (Typical form) | Female ovary and male testes | 45,177 | 1324.66 | 200 | 25,632 |
|  | Female and male brain | 56,385 | 1347.76 | 200 | 23,014 |
|  | Female and male eye | 41,805 | 1493.96 | 200 | 23,013 |
|  | Female and male heart | 28,352 | 1475.56 | 200 | 18,503 |
|  | Female and male hepatopancreas | 35,243 | 1275.35 | 200 | 22,594 |
|  | Female and male kidney | 36,832 | 1413.95 | 200 | 22,606 |
|  | Female and male photophore | 27,673 | 1427.77 | 200 | 22,571 |
|  | Female and male sucker | 43,903 | 1438.38 | 200 | 22,648 |
|  | Female and male tentacle | 36,010 | 1455.85 | 200 | 22,588 |
|  | Female and male testes | 38,574 | 1266.29 | 200 | 24,622 |
| *Sthenoteuthis* sp*.*  (Dwarf form) | Female ovary and male testes | 48,255 | 1476.44 | 200 | 20,844 |
|  | Female and male brain | 56,150 | 1549.72 | 200 | 22,569 |
|  | Female and male eye | 40,463 | 1595.88 | 200 | 20,872 |
|  | Female and male heart | 31,856 | 1547.5 | 200 | 24,737 |
|  | Female and male hepatopancreas | 39,689 | 1363.96 | 200 | 19,782 |
|  | Female and male kidney | 40,881 | 1563.33 | 200 | 31,105 |
|  | Female and male photophore | 26,888 | 1512.01 | 200 | 23,903 |
|  | Female and male sucker | 48,632 | 1531.42 | 200 | 16,809 |
|  | Female and male tentacle | 40,475 | 1553.02 | 200 | 26,308 |
|  | Female and male testes | 37,075 | 1447.34 | 200 | 25,850 |
