# Supplementary material for: Genomes of Two Flying Squid Species Provide Novel Insights into Adaptations of Cephalopods to Pelagic Life
Source: Genomics Proteomics Bioinformatics. 2022 Oct 7;20(6):1053–65. doi: 10.1016/j.gpb.2022.09.009 (PMC10225486; doi:10.1016/j.gpb.2022.09.009)
Supplement: Supplementary Table S7 [file mmc15.docx]

**Table S7**  **The predicted genome completeness of the two *Sthenoteuthis* species** **with metazoa_odb10**

|  | ***S. oualaniensis*** | |  | ***Sthenoteuthis* sp.** | |
| --- | --- | --- | --- | --- | --- |
|  | **Number** | **Percentage (%)** |  | **Number** | **Percentage (%)** |
| Total BUSCO groups searched | 954 | 100 |  | 954 | 100 |
| Complete BUSCOs (C) | 853 | 89.4 |  | 893 | 93.6 |
| Complete and single-copy BUSCOs (S) | 580 | 60.8 |  | 441 | 46.2 |
| Complete and duplicated BUSCOs (D) | 273 | 28.6 |  | 452 | 47.4 |
| Fragmented BUSCOs (F) | 28 | 2.9 |  | 19 | 2.0 |
| Missing BUSCOs (M) | 73 | 7.7 |  | 42 | 4.4 |
