# Supplementary material for: Genomes of Two Flying Squid Species Provide Novel Insights into Adaptations of Cephalopods to Pelagic Life
Source: Genomics Proteomics Bioinformatics. 2022 Oct 7;20(6):1053–65. doi: 10.1016/j.gpb.2022.09.009 (PMC10225486; doi:10.1016/j.gpb.2022.09.009)
Supplement: Supplementary Table S8 [file mmc16.docx]

**Table S8 Transposable elements information for the genome of *S. oualaniensis***

|  | **Repbase TEs** | | **TE proteins** | | ***De novo*** | | **Combined TEs** | |
| --- | --- | --- | --- | --- | --- | --- | --- | --- |
| Type | Length (Bp) | Percent  in genome | Length (Bp) | Percent  in genome | Length (Bp) | Percent  in genome | Length (Bp) | Percent  in genome |
| DNA | 558,511,598 | 9.81 | 12,494,633 | 0.22 | 968,594,716 | 17.01 | 1,450,568,773 | 25.47 |
| LINE | 393,736,244 | 6.91 | 25,155,9979 | 4.42 | 1,119,402,772 | 19.66 | 1,255,803,973 | 22.05 |
| SINE | 13,618,899 | 0.24 | 0 | 0 | 56,359,932 | 0.99 | 69,532,719 | 1.22 |
| LTR | 15,015,9521 | 2.64 | 30,903,245 | 0.54 | 320,972,901 | 5.64 | 446,387,651 | 7.84 |
| Other | 908,764 | 0.02 | 0 | 0 | 0 | 0 | 908,764 | 0.02 |
| Simple repeat | 0 | 0 | 0 | 0 | 360,628,649 | 6.33 | 360,628,649 | 6.33 |
| Unknown | 0 | 0 | 0 | 0 | 506,285,926 | 8.89 | 506,285,926 | 8.89 |
| Total | 89,667,3423 | 15.75 | 294,809,500 | 5.18 | 2,536,051,111 | 44.53 | 3,069,530,109 | 53.90 |
