# Supplementary material for: Genomes of Two Flying Squid Species Provide Novel Insights into Adaptations of Cephalopods to Pelagic Life
Source: Genomics Proteomics Bioinformatics. 2022 Oct 7;20(6):1053–65. doi: 10.1016/j.gpb.2022.09.009 (PMC10225486; doi:10.1016/j.gpb.2022.09.009)
Supplement: Supplementary Table S9 [file mmc17.docx]

**Table S9 Transposable elements information for the genome of *Sthenoteuthis* sp.**

|  | **Repbase TEs** | | **TE proteins** | | ***De novo*** | | **Combined TEs** | |
| --- | --- | --- | --- | --- | --- | --- | --- | --- |
| Type | Length (Bp) | Percent  in genome | Length (Bp) | Percent  in genome | Length (Bp) | Percent  in genome | Length (Bp) | Percent  in genome |
| DNA | 391,738,501 | 6.93 | 4267,081 | 0.08 | 640,298,298 | 11.33 | 798,296,744 | 14.13 |
| LINE | 275,255,583 | 4.87 | 204,179,672 | 3.61 | 424,697,601 | 7.52 | 565,292,718 | 10.00 |
| SINE | 6,468,219 | 0.11 | 0 | 0 | 17,205,635 | 0.30 | 23,645,088 | 0.42 |
| LTR | 99,762,736 | 1.77 | 20,082,106 | 0.36 | 65,053,932 | 1.15 | 153,438,658 | 2.72 |
| Other | 753,396 | 0.01 | 0 | 0 | 0 | 0 | 753,396 | 0.01 |
| Simple repeat | 0 | 0 | 0 | 0 | 14,611,724 | 25.86 | 14,611,724 | 25.86 |
| Unknown | 0 | 0 | 0 | 0 | 1,294,749,419 | 22.91 | 1,294,749,419 | 22.91 |
| Total | 601,351,127 | 10.64 | 228,510,191 | 4.04 | 2,278,563,325 | 40.32 | 2,394,387,829 | 42.37 |
