# Supplementary material for: Genomes of Two Flying Squid Species Provide Novel Insights into Adaptations of Cephalopods to Pelagic Life
Source: Genomics Proteomics Bioinformatics. 2022 Oct 7;20(6):1053–65. doi: 10.1016/j.gpb.2022.09.009 (PMC10225486; doi:10.1016/j.gpb.2022.09.009)
Supplement: Supplementary Table S10 [file mmc18.docx]

**Table S10 66 positively selected genes for the purpleback flying squids' clades with *P* < 0.01**

| **Gene ID in *S.oualaniensis*** | **Gene ID in *Sthenoteuthis sp.*** | **Gene name** | ***P* value** |
| --- | --- | --- | --- |
| Stoua_s03870_0001 | Ssto026870 | *syncrip* | 0 |
| Stoua_s02768_0003 | Ssto026306 | *atm* | 0 |
| Stoua_s00320_0007 | Ssto024424 | *bmp4* | 0 |
| Stoua_s03733_0006 | Ssto024280 | *xpo7* | 0 |
| Stoua_s01866_0007 | Ssto020258 | *sf3a3* | 0 |
| Stoua_s00815_0001 | Ssto013605 | *nfs1* | 0 |
| Stoua_s00407_0007 | Ssto004645 | *cachd1* | 0 |
| Stoua_s00590_0007 | Ssto014662 | *ars2* | 0 |
| Stoua_s06214_0003 | Ssto011351 | *unc80* | 0 |
| Stoua_s00265_0007 | Ssto027203 | *ascc3* | 0 |
| Stoua_s00393_0008 | Ssto004174 | *prmt7* | 0 |
| Stoua_s02684_0001 | Ssto002215 | *mgea5* | 2.22E-16 |
| Stoua_s06242_0001 | Ssto013541 | *nedd4* | 2.22E-16 |
| Stoua_s00282_0004 | Ssto024669 | *ippk* | 1.55E-15 |
| Stoua_s00564_0008 | Ssto014605 | *ambra1* | 1.55E-15 |
| Stoua_s06540_0001 | Ssto000868 | *ino80d* | 5.33E-15 |
| Stoua_s09115_0002 | Ssto022041 | *znf839* | 5.55E-15 |
| Stoua_s02772_0005 | Ssto027163 | *rabgap1* | 5.02E-14 |
| Stoua_s00009_0041 | Ssto002933 | *dpys* | 1.86E-13 |
| Stoua_s01148_0003 | Ssto025284 | *usp9x* | 8.87E-13 |
| Stoua_s04416_0003 | Ssto008192 | *insig2* | 2.28E-12 |
| Stoua_s01214_0001 | Ssto006769 | *bora* | 2.74E-12 |
| Stoua_s09069_0001 | Ssto007035 | *zfyve26* | 4.80E-12 |
| Stoua_s02175_0003 | Ssto024903 | *mapre1* | 7.10E-10 |
| Stoua_s05524_0002 | Ssto027228 | *fgfr1op* | 7.17E-10 |
| Stoua_s00009_0044 | Ssto005062 | *cd164* | 2.45E-09 |
| Stoua_s10779_0002 | Ssto015283 | *slc25a32* | 5.63E-09 |
| Stoua_s08935_0001 | Ssto025463 | *wdr3* | 1.34E-08 |
| Stoua_s06966_0009 | Ssto004631 | *dtl* | 1.97E-08 |
| Stoua_s02706_0004 | Ssto020642 | *mmaa* | 2.81E-08 |
| Stoua_s00510_0002 | Ssto002166 | *c7orf26* | 6.76E-08 |
| Stoua_s01049_0001 | Ssto021889 | *insr* | 7.11E-08 |
| Stoua_s03610_0005 | Ssto017106 | *ndufa6* | 1.39E-07 |
| Stoua_s01870_0004 | Ssto005171 | *mnt* | 1.62E-07 |
| Stoua_s01367_0006 | Ssto001318 | *ndufs7* | 4.10E-07 |
| Stoua_s20486_0001 | Ssto004173 | *timm17b* | 5.84E-07 |
| Stoua_s03357_0002 | Ssto025730 | *tjp1* | 1.10E-06 |
| Stoua_s00368_0001 | Ssto026187 | *lasp* | 2.73E-06 |
| Stoua_s00185_0019 | Ssto012363 | *pacs2* | 3.57E-06 |
| Stoua_s01496_0003 | Ssto006657 | *fam117b* | 7.23E-06 |
| Stoua_s03049_0002 | Ssto018770 | *mms22l* | 1.03E-05 |
| Stoua_s00289_0006 | Ssto015278 | *gphn* | 1.92E-05 |
| Stoua_s16128_0001 | Ssto021882 | *chmp1a* | 5.32E-05 |
| Stoua_s06687_0004 | Ssto020141 | *lamtor2* | 5.89E-05 |
| Stoua_s00098_0009 | Ssto025582 | *eef2k* | 6.35E-05 |
| Stoua_s00446_0004 | Ssto015057 | *exosc7* | 6.54E-05 |
| Stoua_s00274_0006 | Ssto015772 | *anapc5* | 6.96E-05 |
| Stoua_s04160_0002 | Ssto002098 | *srrd* | 7.78E-05 |
| Stoua_s02233_0003 | Ssto002765 | *gcc1* | 0.000119295 |
| Stoua_s14705_0001 | Ssto023421 | *znf280c* | 0.000150344 |
| Stoua_s00560_0006 | Ssto020147 | *copa* | 0.000185983 |
| Stoua_s08936_0001 | Ssto008748 | *sf3b3* | 0.000213642 |
| Stoua_s01367_0008 | Ssto001321 | *bzw2* | 0.000500633 |
| Stoua_s01836_0009 | Ssto019519 | *lsm5* | 0.000566332 |
| Stoua_s01685_0005 | Ssto003270 | *none* | 0.001110618 |
| Stoua_s01815_0003 | Ssto000776 | *dnpep* | 0.001140785 |
| Stoua_s02490_0003 | Ssto005680 | *ufl1* | 0.001436811 |
| Stoua_s00848_0004 | Ssto019063 | *tnip2* | 0.001614129 |
| Stoua_s01722_0006 | Ssto019849 | *pgk1* | 0.001957393 |
| Stoua_s00023_0014 | Ssto003187 | *sli* | 0.002458533 |
| Stoua_s10549_0001 | Ssto024230 | *auh* | 0.002882639 |
| Stoua_s21342_0001 | Ssto022797 | *paqr3* | 0.003196648 |
| Stoua_s04858_0002 | Ssto015809 | *pgs1* | 0.003559868 |
| Stoua_s01374_0001 | Ssto001510 | *bsk* | 0.006474727 |
| Stoua_s00354_0012 | Ssto025693 | *calr* | 0.006717453 |
| Stoua_s13358_0001 | Ssto025302 | None | 0.006969673 |
