# Supplementary material for: Genomes of Two Flying Squid Species Provide Novel Insights into Adaptations of Cephalopods to Pelagic Life
Source: Genomics Proteomics Bioinformatics. 2022 Oct 7;20(6):1053–65. doi: 10.1016/j.gpb.2022.09.009 (PMC10225486; doi:10.1016/j.gpb.2022.09.009)
Supplement: Supplementary Table S11 [file mmc19.docx]

**Table S11**  **Terms from the Function Ontology with FDR < 0.05 for the 66 PSGs of the two *Sthenoteuthis* lineage. The top 20 terms were listed**

| **GO ID** | **GO term** | **Cluster frequency** | **Genome frequency of use** | **FDR** |
| --- | --- | --- | --- | --- |
| Phosphoglycerate kinase activity | GO:0004618 | 2 out of 83 genes | 2 out of 28898 genes | < 1.00E–4 |
| Phosphomannomutase activity | GO:0004615 | 2 out of 83 genes | 2 out of 28898 genes | < 1.00E–4 |
| Elongation factor-2 kinase activity | GO:0004686 | 2 out of 83 genes | 2 out of 28898 genes | < 1.00E–4 |
| Cysteine desulfurase activity | GO:0031071 | 2 out of 83 genes | 2 out of 28898 genes | < 1.00E–4 |
| Inositol pentakisphosphate 2-kinase activity | GO:0035299 | 2 out of 83 genes | 2 out of 28898 genes | < 1.00E–4 |
| P-P-bond-hydrolysis-driven protein transmembrane transporter activity | GO:0015450 | 2 out of 83 genes | 5 out of 28898 genes | < 1.00E–4 |
| Phosphotransferase activity, carboxyl group as acceptor | GO:0016774 | 2 out of 83 genes | 5 out of 28898 genes | < 1.00E–4 |
| Protein transmembrane transporter activity | GO:0008320 | 2 out of 83 genes | 8 out of 28898 genes | < 1.00E–4 |
| Peptide transmembrane transporter activity | GO:1904680 | 2 out of 83 genes | 8 out of 28898 genes | < 1.00E–4 |
| Macromolecule transmembrane transporter activity | GO:0022884 | 2 out of 83 genes | 10 out of 28898 genes | 0.0018 |
| Phosphatidylinositol-3-phosphate binding | GO:0032266 | 2 out of 83 genes | 9 out of 28898 genes | 0.002 |
| Amide transmembrane transporter activity | GO:0042887 | 2 out of 83 genes | 12 out of 28898 genes | 0.0029 |
| Insulin-like growth factor binding | GO:0005520 | 2 out of 83 genes | 11 out of 28898 genes | 0.0031 |
| Calmodulin-dependent protein kinase activity | GO:0004683 | 2 out of 83 genes | 11 out of 28898 genes | 0.0033 |
| Transferase activity, transferring phosphorus-containing groups | GO:0016772 | 14 out of 83 genes | 1842 out of 28898 genes | 0.004 |
| Protein serine/threonine kinase activity | GO:0004674 | 6 out of 83 genes | 390 out of 28898 genes | 0.0094 |
| Phosphatidylinositol phosphate binding | GO:1901981 | 2 out of 83 genes | 15 out of 28898 genes | 0.010 |
| Quinone binding | GO:0048038 | 2 out of 83 genes | 19 out of 28898 genes | 0.011 |
| Growth factor binding | GO:0019838 | 2 out of 83 genes | 18 out of 28898 genes | 0.012 |
| Kinase activity | GO:0016301 | 12 out of 83 genes | 1524 out of 28898 genes | 0.012 |
