# Supplementary material for: Genomes of Two Flying Squid Species Provide Novel Insights into Adaptations of Cephalopods to Pelagic Life
Source: Genomics Proteomics Bioinformatics. 2022 Oct 7;20(6):1053–65. doi: 10.1016/j.gpb.2022.09.009 (PMC10225486; doi:10.1016/j.gpb.2022.09.009)
Supplement: Supplementary Table S12 [file mmc20.docx]

**Table S12**  **Terms from the Process Ontology with FDR < 0.05 for the 66 PSGs of the two *Sthenoteuthis* lineage. The top 20 terms were listed**

| **GO ID** | **GO term** | **Cluster frequency** | **Genome frequency of use** | **FDR** |
| --- | --- | --- | --- | --- |
| [2Fe-2S] cluster assembly | GO:0044571 | 2 out of 59 genes | 2 out of 19047 genes | < 1.00E–4 |
| GDP-mannose biosynthetic process | GO:0009298 | 2 out of 59 genes | 2 out of 19047 genes | < 1.00E–4 |
| Molybdopterin cofactor biosynthetic process | GO:0032324 | 2 out of 59 genes | 2 out of 19047 genes | < 1.00E–4 |
| Replicative senescence | GO:0090399 | 2 out of 59 genes | 2 out of 19047 genes | < 1.00E–4 |
| Aging | GO:0007568 | 2 out of 59 genes | 5 out of 19047 genes | 0.013 |
| GDP-mannose metabolic process | GO:0019673 | 2 out of 59 genes | 3 out of 19047 genes | 0.013 |
| Cytokinesis | GO:0000910 | 3 out of 59 genes | 26 out of 19047 genes | 0.014 |
| Histone phosphorylation | GO:0016572 | 2 out of 59 genes | 6 out of 19047 genes | 0.016 |
| Cell aging | GO:0007569 | 2 out of 59 genes | 3 out of 19047 genes | 0.016 |
| Intracellular protein transport | GO:0006886 | 7 out of 59 genes | 418 out of 19047 genes | 0.026 |
| Response to abiotic stimulus | GO:0009628 | 3 out of 59 genes | 48 out of 19047 genes | 0.031 |
| Nucleotide-sugar biosynthetic process | GO:0009226 | 2 out of 59 genes | 11 out of 19047 genes | 0.035 |
| Microtubule anchoring | GO:0034453 | 2 out of 59 genes | 13 out of 19047 genes | 0.036 |
| Cellular macromolecule localization | GO:0070727 | 7 out of 59 genes | 525 out of 19047 genes | 0.038 |
| Protein import into mitochondrial matrix | GO:0030150 | 2 out of 59 genes | 17 out of 19047 genes | 0.038 |
| Response to ionizing radiation | GO:0010212 | 2 out of 59 genes | 13 out of 19047 genes | 0.039 |
| Cellular protein localization | GO:0034613 | 7 out of 59 genes | 525 out of 19047 genes | 0.040 |
| Nucleotide-sugar metabolic process | GO:0009225 | 2 out of 59 genes | 17 out of 19047 genes | 0.040 |
| Prosthetic group metabolic process | GO:0051189 | 2 out of 59 genes | 16 out of 19047 genes | 0.042 |
| Molybdopterin cofactor metabolic process | GO:0043545 | 2 out of 59 genes | 16 out of 19047 genes | 0.044 |
